# Role of meteorological factors in the transmission of SARS-CoV-2 in the United States

**DOI:** 10.1038/s41467-021-23866-7

**Published:** 2021-06-14

**Authors:** Yiqun Ma, Sen Pei, Jeffrey Shaman, Robert Dubrow, Kai Chen

**Affiliations:** 1 Department of Environmental Health Sciences, Yale School of Public Health, New Haven, CT USA; 2 Yale Center on Climate Change and Health, Yale School of Public Health, New Haven, CT USA; 3 Department of Environmental Health Sciences, Mailman School of Public Health, Columbia University, New York, NY USA

**Keywords:** SARS-CoV-2, Ecological epidemiology, Epidemiology, Risk factors

## Abstract

Improved understanding of the effects of meteorological conditions on the transmission of SARS-CoV-2, the causative agent for COVID-19 disease, is needed. Here, we estimate the relationship between air temperature, specific humidity, and ultraviolet radiation and SARS-CoV-2 transmission in 2669 U.S. counties with abundant reported cases from March 15 to December 31, 2020. Specifically, we quantify the associations of daily mean temperature, specific humidity, and ultraviolet radiation with daily estimates of the SARS-CoV-2 reproduction number (*R*_*t*_) and calculate the fraction of *R*_*t*_ attributable to these meteorological conditions. Lower air temperature (within the 20–40 °C range), lower specific humidity, and lower ultraviolet radiation were significantly associated with increased *R*_*t*_. The fraction of *R*_*t*_ attributable to temperature, specific humidity, and ultraviolet radiation were 3.73% (95% empirical confidence interval [eCI]: 3.66–3.76%), 9.35% (95% eCI: 9.27–9.39%), and 4.44% (95% eCI: 4.38–4.47%), respectively. In total, 17.5% of *R*_*t*_ was attributable to meteorological factors. The fractions attributable to meteorological factors generally were higher in northern counties than in southern counties. Our findings indicate that cold and dry weather and low levels of ultraviolet radiation are moderately associated with increased SARS-CoV-2 transmissibility, with humidity playing the largest role.

## Abstract (web summary)

The role of meteorological factors in SARS-COV-2 transmission is not well understood. Here, the authors use county-level data from the United States to the end of 2020 and find evidence of a moderate association between increased transmissibility and cold, dry weather and low ultraviolet radiation.

## Introduction

Since first detected, the novel severe acute respiratory syndrome coronavirus 2 (SARS-CoV-2), the causative agent of coronavirus disease 2019 (COVID-19), has produced a major global pandemic. As of March 20, 2021, ~29.8 million COVID-19 cases and 542 thousand deaths had been reported in the U.S.^1^, more than any other country. The decreased stability of SARS-CoV-2 in warmer temperatures, higher humidity, and simulated sunlight in laboratory experiments^2–5^, and the documented seasonality of influenza^6,7^ and infections caused by other coronaviruses^8–10^, lead to the hypothesis that lower air temperature, lower humidity, and lower ultraviolet (UV) radiation are associated with increased SARS-CoV-2 transmission. Quantifying this effect on a population level is needed to help inform public health control efforts, including transmission prevention and communication with the public^11^.

Numerous preliminary studies have found either positive or negative associations of air temperature, humidity, and UV radiation with reported COVID-19 case numbers^12–17^. However, given the large number of undocumented SARS-CoV-2 infections^18^, the variations in the lag between infection and symptom onset, and the inconsistent lag between testing and reporting, using daily new confirmed cases may not be optimal for examining meteorological effects^19^. As a result, a few studies have used the reproduction number to estimate SARS-CoV-2 transmissibility^20–22^. One study reported high daily air temperature and high daily relative humidity (RH, the amount of water vapor in the air expressed as a percentage of the amount needed for saturation at a given temperature) to be associated with a reduced daily effective reproduction number (*R*_*e*_, the mean number of new infections caused by a single infected person in a population in which some individuals may no longer be susceptible due to acquired immunity^23^) for SARS-CoV-2 in both China and the U.S.^20^. However, early studies focused on the first few of months of the pandemic found no association between temperature, humidity, or UV radiation and the basic reproduction number (the mean number of new infections caused by a single infected person in a population in which everyone is assumed to be susceptible and no public health measures have been implemented)^21,22^.

Early analyses, in particular, should be interpreted with caution^11^, as the range of temperature, humidity, and UV radiation measurements during the short observation period at the beginning of the pandemic was relatively narrow in most studies^12–15,20–22^, thus limiting the ability to detect associations between these meteorological variables and SARS-CoV-2 transmission. In addition, many previous studies (whether using COVID-19 cases or reproduction number as the outcome) controlled for no or only a few potential confounders^12–16,21,22^, which include other environmental factors, socioeconomic factors, temporal changes in population immunity, and implementation of public health interventions.

Furthermore, although most early studies found an association between air temperature, humidity, or UV radiation and COVID-19 incidence, the fraction of cases or deaths attributable to meteorological conditions remains unclear. One modeling study predicted that as long as most of the population is susceptible to infection, any role of humidity in SARS-CoV-2 transmission would be overwhelmed by the lack of population immunity^24^. This prediction is supported by the rapid transmission of SARS-CoV-2 regardless of climate zone, including warmer locations such as tropical Brazil, India, and southern states in the U.S. during the northern hemisphere summer^1^. In addition, the relative importance of different meteorological factors needs further investigation^25^.

Here we investigate the association between meteorological conditions, i.e., air temperature, specific humidity (SH; the mass of water vapor in a unit mass of moist air [g kg^−1^]), and UV radiation and SARS-CoV-2 transmission, as measured by the reproduction number *R*_*t*_ (the mean number of new infections caused by a single infected person, given the public health measures in place, in a population in which everyone is assumed to be susceptible). In this study, instead of *R*_*e*_, we use *R*_*t*_ to quantify the transmission rate of SARS-CoV-2, which removes the impact of population immunity on disease transmission. We estimate *R*_*t*_ in the 2669 counties with at least 400 cumulative cases as of December 31, 2020 and calculate the fraction of *R*_*t*_ attributable to temperature, SH, or UV radiation, adjusting for a wide range of potential confounders.

## Results

### Distribution of meteorological factors and *R*_*t*_

**Fig. 1:**
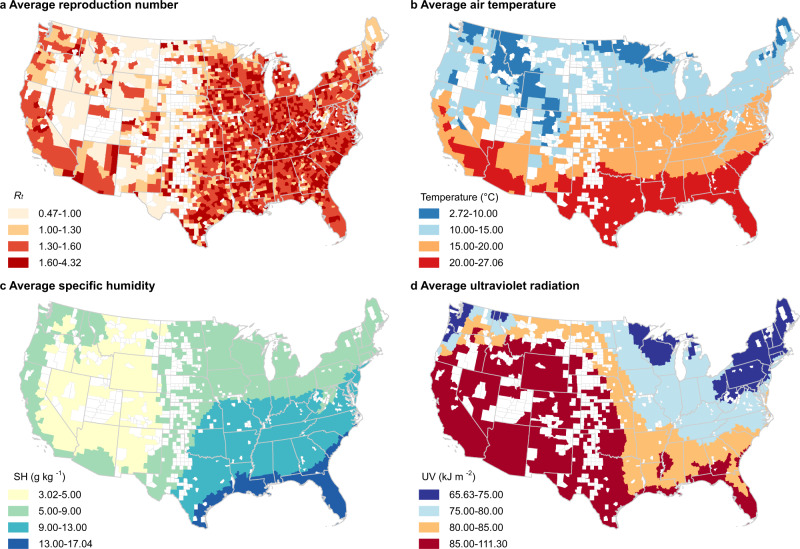
Map of the distribution of reproduction number, air temperature, specific humidity, and ultraviolet radiation in study counties. This figure displays the distribution of key variables averaged over the study period in 2669 U.S. counties. **a** The distribution of the daily reproduction number (*R*_*t*_). **b** The distribution of daily air temperature. **c** The distribution of daily specific humidity (SH). **d** The distribution of daily ultraviolet (UV) radiation. The shapefile in the maps was obtained from the U.S. Census Bureau.

From March 15 to December 31, 2020, a total of 19,430,010 cases of COVID-19 were reported in the 2669 study counties (Supplementary Table 1). We estimated the county-specific *R*_*t*_ using a dynamic metapopulation model informed by human mobility data that represents the transmission of SARS-CoV-2 in the U.S. (see “Methods”). Mean daily *R*_*t*_ averaged over all counties and days during the study period was 1.49 and ranged from 0.45 to 6.62. Daily air temperature, SH, and UV radiation also ranged widely (air temperature: −22.25–39.98 °C; SH: 0.49–22.37 g kg^−1^; UV radiation: 1.68–155.61 kJ m^−2^). Cass County, Indiana had the highest *R*_*t*_ averaged over the study period, and Brewster County, Texas had the lowest (Fig. 1a). Southern counties generally were hotter than northern counties (Fig. 1b). In the eastern U.S., southern counties were more humid than northern counties; western U.S. counties were drier than eastern U.S. counties, with inland western counties generally drier than coastal western counties (Fig. 1c). In the eastern U.S., UV radiation levels were higher in southern counties than in northern counties; western U.S. counties generally were exposed to higher levels of UV radiation than eastern U.S. counties (Fig. 1d).

### Associations between meteorological factors and *R*_*t*_

**Fig. 2:**
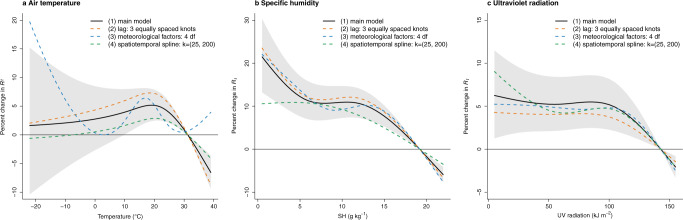
The associations of air temperature, specific humidity, and ultraviolet radiation with *R*_*t*_, under different choices of model. This figure shows the estimated exposure-response curves (meteorological factors vs. percent change in *R*_*t*_) for the associations of air temperature (**a**), specific humidity (SH) (**b**), and ultraviolet (UV) radiation (**c**) with reproduction number (*R*_*t*_) for SARS-CoV-2, with different modelling choices: (1) main model with 95% confidence interval (grey area): tensor product smooths to control for the temporal and spatial variations with a maximum of 30 and 200 knots (k), respectively, and cross-basis terms for meteorological factors, which are defined by natural cubic splines with 3 df for both the exposure-response and lag-response association, with a maximum lag of 13 days; (2) redefine the lag dimension using a natural cubic spline and 3 equally placed internal knots in the log scale; (3) change the df to 4 in the cross-basis terms for meteorological factors in the exposure-response function; (4) change the maximum number of knots to 25 in the flexible natural cubic spline to control time trend in the tensor product smooths.

We estimated the complex non-linear and temporally delayed associations of meteorological factors with SARS-CoV-2 *R*_*t*_ using a generalized additive mixed model adjusting for spatiotemporal variations in *R*_*t*_ and potential measured confounders, described in detail in “Methods”. We then calculated the optimum values of temperature, SH, and UV radiation, which correspond to the lowest *R*_*t*_, between the 1^st^ and the 99^th^ percentiles of the distribution of each meteorological variable. For temperature in the range of 20–40 °C, we found an approximately linear inverse temperature–*R*_*t*_ relationship (Fig. 2a), with lower air temperatures significantly associated with increased transmission of SARS-CoV-2. No significant associations were observed when temperature was below ~10 °C. Compared with the optimum temperature (31.23 °C), a temperature of 20 °C was associated with a 5.15% (95% CI: 2.49–7.88%) increase of *R*_*t*_.

The relationship between SH and *R*_*t*_ was non-linear (Fig. 2b). Higher SH was significantly associated with decreased transmission, except for a stable trend from ~7 to 12 g kg^−1^. Compared with the optimum value (19.21 g kg^−1^), the 1^st^ percentile of the distribution of SH (1.80 g kg^−1^) was associated with a 15.20% (95% CI: 9.65–21.04%) increase of *R*_*t*_. UV radiation level was unrelated to SARS-CoV-2 transmission when UV radiation was lower than ~100 kJ m^−2^, but when above this level, an almost linear negative association was observed between UV radiation and *R*_*t*_ (Fig. 2c). A UV radiation level of 100 kJ m^−2^ was associated with a 5.18% (95% CI: 2.26–8.17%) increase of *R*_*t*_ over the optimal level (142.78 kJ m^−2^).

Trends of effect estimates in the lag dimension are shown in Supplementary Fig. 1. Sensitivity analyses showed the estimated relationships between meteorological factors and *R*_*t*_ to be generally consistent under different modeling choices (Fig. 2a–c), except for the temperature curve when the number of degrees of freedom (df) of exposure (meteorological factors) was changed to 4, which could be a result of overfitting (Fig. 2a). The coefficient table of other covariates in the main model is shown in Supplementary Table 2. The *R*^2^ of the main model is 0.514, and the spatial and temporal autocorrelations are insignificant (*P* values = 0.159 and 0.798 for spatial and temporal autocorrelations, respectively) (Supplementary Table 3).

### Fractions of *R*_*t*_ attributable to meteorological factors

Based on the estimated associations of meteorological factors with *R*_*t*_ and daily county-specific *R*_*t*_, we further calculated the fraction of *R*_*t*_ attributable to meteorological factors (i.e., the attributable fraction [AF], which can be interpreted as the fraction of *R*_*t*_ attributable to the deviation of temperature, SH, or UV radiation from the optimum value). Across all 2669 counties over the entire study period, the AF for temperature was 3.73% (95% empirical confidence intervals [eCI]: 3.66–3.76%), the AF for SH was 9.35% (95% eCI: 9.27–9.39%), and the AF for UV radiation was 4.44% (95% eCI: 4.38–4.47%) (Supplementary Table 4). In total, the three meteorological factors contributed to ~17.5% of *R*_*t*_. Compared with the main model, models including each meteorological factor separately resulted in higher AF for temperature and SH, and lower AF for UV radiation (Supplementary Fig. 2).

**Fig. 3:**
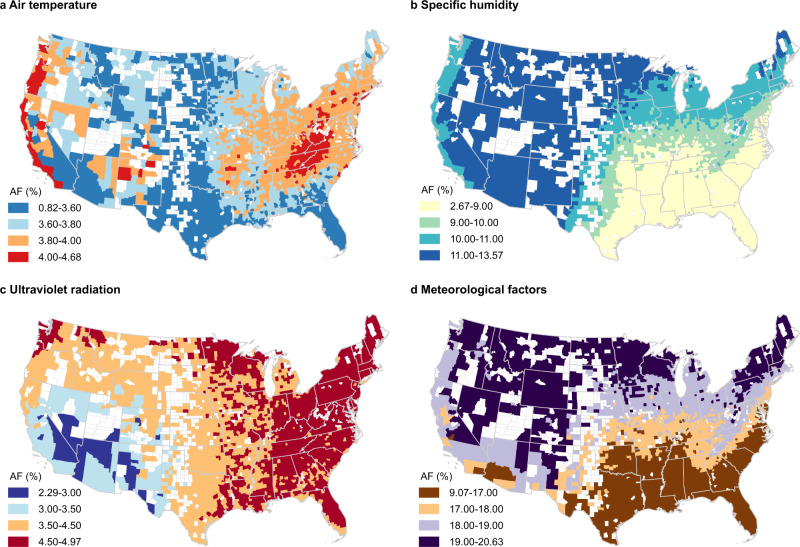
Fractions of *R*_*t*_ attributable to meteorological factors by county. The distribution of the fraction of reproduction number (*R*_*t*_) attributable to temperature (**a**), specific humidity (**b**), ultraviolet radiation (**c**), or the sum of the three meteorological factors (**d**) (i.e., attributable fraction [AF]) in each county. The shapefile in the maps was obtained from the U.S. Census Bureau.

**Fig. 4:**
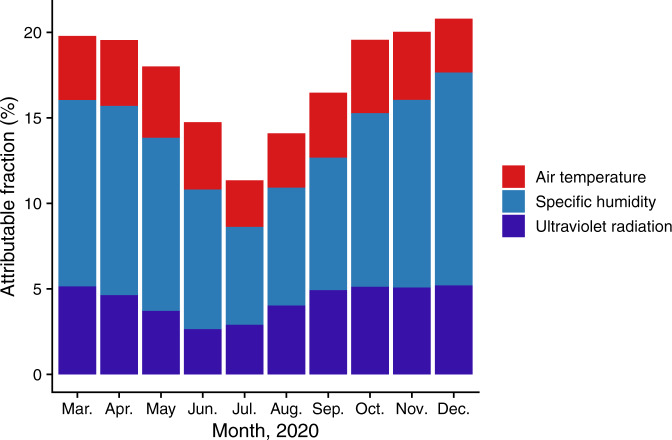
Fractions of *R*_*t*_ attributable to meteorological factors by month. The distribution of the fraction of reproduction number (*R*_*t*_) attributable to temperature, specific humidity, ultraviolet radiation, or the sum of the three meteorological factors (i.e., attributable fraction [AF]) by month in 2020.

The AF for temperature generally was higher in the eastern U.S. and the West Coast than in other regions (Fig. 3a). The AF for SH showed an increasing trend from south to north in the eastern U.S., whereas in the western U.S., the AF for SH was lower in counties in coastal states than in counties in interior states (Fig. 3b). The AF for UV radiation was generally higher in the eastern U.S. than in the western U.S. (Fig. 3c) and was lowest in the southwest. The total AF for all three meteorological factors combined generally was higher in northern counties than in southern counties in the eastern U.S. and was also high in most of the western U.S. (Fig. 3d). Each meteorological factor exhibited the highest AF in winter and the lowest AF in summer (Fig. 4).

Sensitivity analyses indicate that the AF for air temperature, SH, or UV radiation generally remains robust when excluding socioeconomic factors and when additionally adjusting for smoking and obesity prevalence, long-term air pollution, climate zones, or short-term air pollution (Supplementary Table 4).

## Discussion

Using estimated reproduction numbers for 2669 U.S. counties and controlling for temporal and spatial trends and other potential confounders, we assessed the associations of air temperature, SH, and UV radiation with the transmission of SARS-CoV-2 and estimated the fractions of *R*_*t*_ attributable to meteorological factors. We found lower air temperature (within the range of 20–40 °C), lower SH, and lower UV radiation to be significantly associated with increased *R*_*t*_. During the study period, meteorological factors contributed to ~17.5% of *R*_*t*_: 3.73%, 9.35%, and 4.44% of *R*_*t*_ was attributable to the deviation of temperature, SH, and UV radiation from their optimum values, respectively. Meteorological factors in total contributed more to the transmission of SARS-CoV-2 in counties and months with colder and drier weather and lower levels of UV radiation than in counties and months with warmer, more humid weather and higher levels of UV radiation. In December (the month with the lowest temperature, lowest SH, and lowest UV radiation of our study period of March-December), the AF for meteorological factors was 20.8% (Fig. 4). Everything else being equal, we can anticipate the highest AF during the months with colder and drier weather and lower UV radiation in future years.

Associations of lower temperature, lower humidity, and lower amount of UV radiation with increased COVID-19 outcomes have been reported by many previous studies. Many multicity analyses in China reported such negative associations^12,14,15^. For example, using data of daily confirmed case counts from 30 provincial capital cities of China, Liu et al. found that lower temperature and lower absolute humidity were associated with higher COVID-19 case counts^14^. Later, with the rapid spread of COVID-19 around the world, studies in other countries emerged^17,26–29^, and UV radiation was considered in some studies. In the early stages of this pandemic in the U.S., a state-level study of daily COVID-19 case counts observed a declining trend of reported cases with higher UV radiation and increasing temperature up to 52 °F^26^. Based on data from 166 countries worldwide, another study reported that a 1 °C increase in temperature and a 1% increase in RH were associated with a 3.08% and 0.85% reduction in daily new cases, respectively^28^. Another multi-country study provided evidence for a protective role of ultraviolet-B (UVB) radiation in reducing COVID-19 deaths^29^. However, many of these earlier studies were limited by short study periods (e.g. 1–2 months), use of daily confirmed cases or deaths across countries for which there were varying reporting biases, failure to account for the time lag between observed weather conditions and when cases or deaths were recorded, or failure to account for time delays between infection acquisition and case confirmation^19,25^.

By representing the transmissibility of SARS-CoV-2, the estimated daily reproduction number serves as a better outcome than daily case counts. While case counts are subject to the influence of reporting delay and underreporting, which vary across locations and are thus difficult to control, the reproduction number is a direct estimate of the transmission rate of SARS-CoV-2, quantifying the average number of infections caused by one infection in the population. A small number of studies previously analyzed the association between temperature, humidity, or UV radiation and reproduction number^20–22,30^. Wang et al. found that a 1 °C increase in temperature was associated with a reduction in the effective reproduction number of 0.026 in China and 0.020 in the U.S., and a 1% increase in RH was associated with a reduction in the effective reproduction number of 0.0076 in China and 0.0080 in the U.S.^20^. Adnan et al. reported a significant negative association between UV index (a standard measurement of the strength of sunburn-producing UV radiation) and basic reproduction number in major cities of Pakistan^30^. These associations are consistent with our findings but were not supported by two studies in China that examined the basic reproduction number: the first found no association between temperature or UV radiation and SARS-CoV-2 transmission^22^; the second found no association between absolute humidity and SARS-CoV-2 transmission^21^. However, these early studies were limited by short observation periods at the beginning of the pandemic, and they did not account for variations of testing capacity, reporting, human mobility, and population susceptibility in estimating SARS-CoV-2 transmissibility.

We estimated *R*_*t*_ using a dynamic metapopulation model informed by human mobility data. This mechanistic model accounted for unreported infections, reporting delays, and county-to-county movement. Previous estimates of *R*_*e*_ using reported cases did not consider underreporting of infections. Our approach mitigates this limitation by additionally modeling the transmission of unreported infections and estimating the ascertainment rate—the fraction of all infections that are confirmed cases, and the relative contagiousness of those unreported infections. Further, reported incidence is a lagged indicator of disease transmission due to the delay from infection acquisition to laboratory confirmation. We corrected for this lag using a reporting delay model informed by line-list data from the U.S. Lastly, *R*_*e*_ used in previous studies is determined by both the local transmission rate and population susceptibility: ${R}_{e}={R}_{t}\times s$, where *s* is the fraction of the total population susceptible to infection. Analyses using *R*_*e*_ are complicated by the variation of population susceptibility across U.S. counties. To address this issue, we explicitly estimated the population susceptibility in each county, and removed its influence in the calculation of *R*_*t*_^31^ (see “Methods”). The model estimating population susceptibility has been validated against independent seroprevalence study data^31^. Thus, our estimates account for spatial heterogeneity in population immunity.

Another strength of our study was adjustment for a wide range of demographic and socioeconomic factors in the main analysis, as well as for smoking and obesity, air pollution, and climate zone in sensitivity analyses. We also thoroughly controlled for spatially and temporally heterogenous unmeasured confounders, such as implementation of and compliance with public health measures^31^, by simultaneously controlling for temporal and spatial variations (Supplementary Fig. 3) and including smooths of random effects to further account for unmeasured state- and county-level confounding (see “Methods”). This approach accounted for substantial differences in the epidemic curves among states and counties (Supplementary Fig. 4).

Our findings for air temperature and SH are supported by laboratory evidence on the stability of SARS-CoV-2 as a function of temperature and humidity. It has been reported that the virus’ half-life in human nasal mucus and sputum is shorter under conditions of higher temperature and RH than under conditions of lower temperature and RH^2^. Similar findings were reported by other studies testing virus stability in virus transport medium^3^, in aerosols^32^, and on various surfaces^32^. Further, the SARS-CoV-2 half-life was found to be longer at lower temperatures, and at both 22 °C and 27 °C, the half-life decreased as RH increased from 40 to 65% but increased as RH increased from 65 to 85%^33^. This result is roughly consistent with the non-linear relationship between SH and *R*_*t*_ observed in our study (Fig. 2b), in which there was a stable trend of *R*_*t*_ from 7 to 12 g kg^−1^ of SH superimposed on the overall decreasing trend. In addition to being mediated by effects on the virus itself, the associations between temperature and humidity and SARS-CoV-2 transmissibility may be mediated by human airway antiviral defenses. Inhalation of cold and dry air can impair mucociliary clearance, a crucial mechanism for the elimination of inhaled pathogens^34^. Further, during the colder winter months people spend more time indoors, which may facilitate virus transmission^35^. During these months, whether indoors or outdoors, people are exposed to less UV radiation from the sun, which modulates the immune system^36,37^.

UV radiation may affect transmission of SARS-CoV-2 through impacts on the virus and on immune function^25^. It has been shown that higher levels of UV radiation, particularly ultraviolet-C radiation (UV light with wavelengths between 200 and 280 nm), can inactivate RNA viruses^25^. In experimental studies, exposure to simulated sunlight resulted in rapid inactivation of infectious SARS-CoV-2 on different surfaces^4^ and in aerosols^5^. Furthermore, UV radiation can indirectly influence SARS-CoV-2 transmission through its impact on the synthesis of vitamin D and other UV-induced mediators of immune function^36^.

We estimated that a total of ~17.5% of *R*_*t*_ was attributable to the three meteorological factors combined. This estimate is consistent with a previous modelling study, which found that weather (temperature, RH, and UV radiation) explained 17% of the variation in COVID-19 growth rate (i.e., the exponential increase in cases)^38^. We found that SH contributes more to SARS-CoV-2 transmission than temperature, which is consistent with studies of influenza^39,40^. SH is more strongly associated with the observed seasonality of influenza in temperate regions than either temperature or RH^6,39^. In developed countries, such as the U.S., people spend ~90% of their time indoors^41^, especially during winter^35^. Although indoor temperature is usually controlled, indoor humidity generally is not, and closely mirrors outdoor levels^42–44^, perhaps explaining why ambient outdoor SH is more strongly associated with SARS-CoV-2 transmission than ambient outdoor temperature. In addition, the large discrepancy between indoor and outdoor temperature and the high correlation between indoor and outdoor humidity explain why ambient outdoor temperature showed no association with SARS-CoV-2 transmission when lower than 20 °C in the main model (Fig. 2a), but showed a monotonically decreasing trend in the model excluding the other two meteorological factors (Supplementary Fig. 2). However, it remains unclear whether SH (versus temperature) is the causative modulator of SARS-CoV-2 transmission or is simply a useful indicator of the indoor environment and the combined effects of temperature and RH.

In the sensitivity analyses, after adjusting for long-term PM_2.5_, the estimated AF for temperature increased by about 40% (Supplementary Table 4), indicating that long-term PM_2.5_ acted as a confounder for temperature effects. This result is consistent with a recent study that found increased COVID-19 mortality associated with increased long-term exposure to PM_2.5_^45^. In contrast, the fraction of *R*_*t*_ attributable to SH or UV radiation remained stable after adjusting for long-term PM_2.5_. Although it is unclear why long-term PM_2.5_ would serve as a confounder for temperature, but not for SH or UV radiation, this result does suggest that SH and UV radiation are more robust predictors than temperature.

Several limitations of this study should be noted. First, this is an ecological rather than an individual-level study, thus making the study susceptible to the ecological fallacy. Second, due to data limitations, we were unable to explore potential heterogeneity of associations of meteorological factors with *R*_*t*_ for different variants of SARS-CoV-2. Future studies are needed to investigate this potential heterogeneity, as knowledge of differing meteorological impacts across variants may inform prevention strategies.

Our findings indicate that cold and dry weather and low levels of UV radiation are moderately associated with increased SARS-CoV-2 transmissibility in the U.S., with absolute humidity (i.e., SH) playing the greatest role. More extensive public health interventions are needed to mitigate the increased transmissibility of SARS-CoV-2 in winter months.

## Methods

### Data collection

We extracted hourly air temperature and SH from the North America Land Data Assimilation System project^46^, a near real-time dataset with a 0.125° × 0.125° grid resolution. We spatially and temporally averaged these data into daily county-level records. SH is the mass of water vapor in a unit mass of moist air (g kg^−1^). Daily downward UV radiation at the surface, with a wavelength of 0.20–0.44 µm, was extracted from the European Centre for Medium-Range Weather Forecasts ERA5 climate reanalysis^47^.

Other characteristics of each county, including geographic location, population density, demographic structure of the population, socioeconomic factors, proportion of healthcare workers, intensive care unit (ICU) bed capacity, health risk factors, long-term and short-term air pollution, and climate zone were collected from multiple sources. Geographic coordinates, population density, median household income, percent of people older than 60 years, percent Black residents, percent Hispanic residents, percent owner-occupied housing, percent residents aged 25 years and over without a high school diploma, and percent healthcare practitioners or support staff were collected from the U.S. Census Bureau^48^. Total ICU beds in each county were derived from Kaiser Health News^49^. The prevalence of smoking and obesity among adults in each county was obtained from the Robert Wood Johnson Foundation’s 2020 County Health Rankings^50^. We extracted annual PM_2.5_ concentrations in the U.S. from 2014 to 2018 from the 0.01° × 0.01° grid resolution PM_2.5_ estimation provided by the Atmospheric Composition Analysis Group^51^, and calculated average PM_2.5_ levels during this 5-year period for each county to represent long-term PM_2.5_ exposure (Supplementary Fig. 5). Short-term air quality data during the study period, including daily mean PM_2.5_ and daily maximum 8-h O_3_, were obtained from the United States Environmental Protection Agency^52^. We categorized study counties into one of five climate zones based on the guide released by U.S. Department of Energy^53^ (Supplementary Fig. 6).

The county-level COVID-19 case and death data were downloaded from the John Hopkins University Coronavirus Resource Center^1^. The U.S. county-to-county commuting data were available from the U.S. Census Bureau^48^. Daily numbers of inter-county visitors to points of interest (POI) were provided by SafeGraph^54^.

### Data ethics

SafeGraph utilizes data from mobile applications of which users optionally consent to provide their anonymous location data.

### Estimation of reproduction number

We estimated the daily reproduction number (*R*_*t*_) in all 3142 U.S. counties using a dynamic metapopulation model informed by human mobility data^31,55^. *R*_*t*_ is the mean number of new infections caused by a single infected person, given the public health measures in place, in a population in which everyone is assumed to be susceptible. In the metapopulation model, two types of movement were considered: daily work commuting and random movement. During the daytime, some commuters travel to a county other than their county of residence, where they work and mix with the populations of that county; after work, they return home and mix with individuals in their home, residential county. Apart from regular commuting, a fraction of the population in each county, assumed to be proportional to the number of inter-county commuters, travels for purposes other than work. As the population present in each county is different during daytime and night-time, we modelled the transmission dynamics of COVID-19 separately for these two time periods, each depicted by a set of ordinary differential equations (Supplementary Notes).

To account for case underreporting, we explicitly simulated reported and unreported infections, for which separate transmission rates were defined. Recent studies from several countries indicate that asymptomatic cases of COVID-19, which are typically unreported, are less contagious than symptomatic cases^56–59^. Studies on the early transmission of SARS-CoV-2 in China^18^ and the U.S.^60^ also showed that undocumented infections are less transmissible than documented infections.

In order to reflect the spatiotemporal variation of disease transmission rate and reporting, we allowed transmission rates and ascertainment rates to vary across counties and to change over time. The transmission model simulated daily confirmed cases and deaths for each county. To map infections to deaths, we used an age-stratified infection fatality rate (IFR)^61^ and computed the weekly IFR for each county as a weighted average using state-level age structure of confirmed cases reported by the U.S. Centers for Disease Control and Prevention. We further adjusted for reporting lags using an observational delay model informed by a U.S. line-list COVID-19 data record^62^.

For the period prior to March 15, 2020, we used commuting data from the U.S. census survey to prescribe the inter-county movement in the transmission model^48^. Starting March 15, the census survey data are no longer representative due to changes in mobility behavior following the implementation of non-pharmaceutical interventions. We, therefore, used estimates of the reduction of inter-county visitors to POI (e.g., restaurants, stores, etc.) from SafeGraph^54^ to account for the change in inter-county movement on a county-by-county basis. Because there is no direct relationship between population-level mobility patterns and COVID-19 transmission rates^63^, we did not model local transmission rate as a function of inter-county mobility. Instead, the SafeGraph data were only used to inform the change of population mixing across counties.

To infer key epidemiological parameters, we fitted the transmission model to county-level daily cases and deaths reported from March 15, 2020 to December 31, 2020. The estimated reproduction number was computed as follows:

1 $${R}_{t}=\beta D\left[\alpha +\left(1-\alpha \right)\mu \right],$$

where *β* is the county-specific transmission rate, *μ* is the relative transmissibility of unreported infections, *α* is the county-specific ascertainment rate, and *D* is the average duration of infectiousness. Note $\beta$ and $\alpha$ were defined for each county separately and were allowed to vary over time. Unlike previous studies using effective reproduction number

2 $${R}_{e}=\beta D\left[\alpha +\left(1-\alpha \right)\mu \right]s,$$

where *s* is the estimated local population susceptibility, we used reproduction number *R*_*t*_ to exclude the influence of population susceptibility on disease transmission rate.

*D*, $\mu$, $Z$ (the average latency period from infection to contagiousness), and a multiplicative factor adjusting random movement ($\theta$) were randomly drawn from the posterior distributions inferred from case data through March 13, 2020^60^: $D=3.56$ (3.21–3.83), $\mu =0.64$ (0.56–0.70), $Z=3.59$ (95% CI: 3.28–3.99), and $\theta =0.15$ (0.12–0.17). $Z$ and $\theta$ are used in ordinary differential equations used to model transmission dynamics (Supplementary Notes).

The daily transmission rate $\beta$ and ascertainment rate $\alpha$ were estimated sequentially for each county using the ensemble adjustment Kalman filter (EAKF)^64^. Specifically, parameters ${\beta }_{i}$ and ${\alpha }_{i}$ for county $i$ were updated each day using incidence and death data. We used the estimates on day $t-1$ as the prior parameters on day $t$, and then updated the priors to posteriors using the EAKF and observations. The posteriors are the estimated parameter values on day $t$. To ensure a smooth parameter estimation, we imposed a $\pm 30 \%$ limit on the daily change of parameters ${\beta }_{i}$ and ${\alpha }_{i}$. Other smoothing constraints were tested and the results were similar. To avoid possible inaccurate estimation for counties with few cases, we inferred *R*_*t*_ in the 2669 U.S. counties with at least 400 cumulative confirmed cases as of December 31, 2020 (Supplementary Fig. 7).

### Statistical analysis

All statistical analyses were conducted with R software (version 3.6.1) using the *mgcv* and *dlnm* packages.

### Association between meteorological factors and *R*_*t*_

Given the potential non-linear and temporally delayed effects of meteorological factors, a distributed lag non-linear model^65^ combined with generalized additive mixed models^66^ was applied to estimate the associations of daily mean temperature, daily mean SH, and daily mean UV radiation with SARS-CoV-2 *R*_*t*_. To quantify the total contribution, independent effects, and relative importance of meteorological factors (i.e., temperature, SH, and UV radiation), we included all three variables in the same model. To reduce collinearity, we used cross-basis terms rather than the raw variables (Supplementary Tables 5–6). The full model can be expressed as:

3 $$\log (E({{{R}}}_{i,j,t}))=	\alpha +te(s({{\rm{latitude}}}_{i}{,{\rm{longitude}}}_{i},{\rm{k}}=200),s({{\rm{time}}}_{t},{\rm{k}}=30))+{\rm{cb}}.{\rm{temperature}}+{\rm{cb}}.{\rm{SH}}+ {\rm{cb}}.{\rm{UV}}\\ 	+{\beta }_{1}({\rm{population}}\,{\rm{density}}_{i})+{\beta }_{2}({\rm{percent}}\,{\rm{Black}}\,{\rm{residents}}_{i})+{\beta }_{3}({\rm{percent}}\,{\rm{Hispanic}}\,{\rm{residents}}_{i})\\ 	 +{\beta }_{4}({\rm{percent}}\,{\rm{people}}\,{\rm{older}}\,{\rm{than}}\,60\,{\rm{years}}_{i})+{\beta }_{5}({\rm{median}}\,{\rm{household}}\,{\rm{income}}_{i})\\ 	+{\beta }_{6}({\rm{percent}}\,{\rm{owner}}-{\rm{occupied}}\,{\rm{housing}}_{i})\\ 	 +{\beta }_{7}({\rm{percent}}\,{\rm{residents}}\,{\rm{older}}\,{\rm{than}}\,25\,{\rm{years}}\,{\rm{without}}\,{\rm{a}}\,{\rm{high}}\,{\rm{school}}\,{\rm{diploma}}_{i})\\ 	 +{\beta }_{8}({\rm{number}}\,{\rm{of}}\,{\rm{ICU}}\,{\rm{beds}}\,{\rm{per}}\,10,000\,{\rm{people}}_{i})+{\beta }_{9}({\rm{percent}}\,{\rm{healthcare}}\,{\rm{workers}}_{i})\\ 	 \quad \, {\beta }_{10}({\rm{day}}\,{\rm{when}}\,100\,{\rm{cumulative}}\,{\rm{cases}}\,{\rm{per}}\,100,000\,{\rm{people}}\,{\rm{was}}\,{\rm{reached}}_{i})+{re}({\rm{county}}_{i})+{re}({\rm{state}}_{j})$$

where *E*(*R*_*i,j,t*_) refers to the expected *R*_*t*_ in county *i*, state *j*, on day *t*, and *α* is the intercept. Given the distribution of *R*_*t*_ in our data close to a lognormal distribution (Supplementary Fig. 8), we used log-transformed *R*_*t*_ as the outcome variable, and the Gaussian family in the model. A thin plate spline with a maximum of 200 knots was used to control the coordinates of the centroid of each county; the time trend was controlled by a flexible natural cubic spline over the range of study dates with a maximum of 30 knots; due to the unique pattern of the non-linear time trend of *R*_*t*_ in each county (Supplementary Fig. 4), we constructed tensor product smooths (*te*) of the splines of geographical coordinates and time, to better control for the temporal and spatial variations (Supplementary Fig. 3).

Cb.temperature, cb.SH, and cb.UV are cross-basis terms for the mean air temperature, mean SH and mean UV radiation, respectively. We modeled exposure-response associations (meteorological factors vs. percent change in *R*_*t*_) using a natural cubic spline with 3 degrees of freedom (df) and modeled the lag-response association using a natural cubic spline with an intercept and 3 df with a maximum lag of 13 days. We adjusted for county-level characteristics, including population density, percent Black residents, percent Hispanic residents, percent people older than 60 years, median household income, percent owner-occupied housing, percent residents older than 25 years without a high school diploma, number of ICU beds per 10,000 people, and percent healthcare workers, given their potential relationship with SARS-CoV-2 transmission^67–70^. Day when 100 cumulative cases per 100,000 people was reached in each county was used to approximate local epidemic stage^45^ (Supplementary Fig. 9). The random effects of state and county were modeled by parametric terms penalized by a ridge penalty (*re*), to further control for unmeasured state- and county-level confounding. Residual plots were used to diagnose the model (Supplementary Fig. 10). In additional analyses, we included air temperature, SH, and UV radiation in separate models (Supplementary Fig. 2).

Based on the estimated exposure-response curves, between the 1^st^ and the 99^th^ percentiles of the distribution of air temperature, SH, and UV radiation, we determined the value of exposure associated with the lowest relative risk of *R*_*t*_ to be the optimum temperature, the optimum SH, or the optimum UV radiation, respectively. The natural cubic spline functions of the exposure-response relationship were then re-centered with the optimum values of meteorological factors as reference values. We report the cumulative relative risk of *R*_*t*_ associated with daily temperature, SH, or UV radiation exposure in the previous two weeks (0– 13 lag days) as the percent changes in *R*_*t*_ when comparing the daily exposure with the optimum reference values (i.e., the cumulative relative risk of *R*_*t*_ equals one and the percent change in *R*_*t*_ equals zero when the temperature, SH, or UV radiation exposure is at its optimum value).

### Attribution of *R*_*t*_ to meteorological factors

We used the optimum value of temperature, SH, or UV radiation as the reference value for calculating the fraction of *R*_*t*_ attributable to each meteorological factor; i.e., the attributable fraction (AF). For these calculations, we assumed that the associations of meteorological factors with *R*_*t*_ were consistent across the counties. For each day in each county, based on the cumulative lagged effect (cumulative relative risk) corresponding to the temperature, SH, or UV radiation of that day, we calculated the attributable *R*_*t*_ in the current and next 13 days, using a previously established method^71^. Specifically, in a given county, the *R*_*t*_ attributable to a meteorological factor (*x*_*t*_) for a given day *t* was defined as the attributable absolute excess of *R*_*t*_ (*AE*_*x,t*_, the excess reproduction number on day *t* attributable to the deviation of temperature or SH from the optimum value) and the attributable fraction of *R*_*t*_ (*AF*_*x*,_, the fraction of *R*_*t*_ attributable to the deviation of the meteorological factor from its optimum value), each accumulated over the current and next 13 days. The formulas can be expressed as:

4 $${{AF}}_{x,t}=1-{\rm{exp }}\left(-\mathop{\sum }\limits_{l=0}^{13}{\beta }_{{x}_{t},l}\right)$$

5 $${{AE}}_{x,t}={{AF}}_{x,t}\times \mathop{\sum }\limits_{l=0}^{13}\frac{{n}_{t+1}}{13+1},$$

where *n*_*t*_ is the *R*_*t*_ on day *t*, and ${\sum }_{l=0}^{13}{\beta }_{{x}_{t},l}$ is the overall cumulative log-relative risk for exposure *x*_*t*_ on day *t* obtained by the exposure-response curves re-centered on the optimum values. Then, the total absolute excess of *R*_*t*_ attributable to temperature, SH, or UV radiation in each county was calculated by summing the absolute excesses of all days during the study period, and the attributable fraction was calculated by dividing the total absolute excess of *R*_*t*_ for the county by the sum of the *R*_*t*_ of all days during the study period for the county. The attributable fraction for the 2669 counties combined was calculated in a similar manner at the national level. We derived the 95% eCI for the attributable absolute excess and attributable fraction by 1000 Monte Carlo simulations^71^. The total fraction of *R*_*t*_ attributable to meteorological factors was the sum of the attributable fraction for temperature, SH, and UV radiation. We also calculated the attributable fractions by month in the study period.

### Sensitivity analyses

We conducted several sensitivity analyses to test the robustness of our results: (a) the lag dimension was redefined using a natural cubic spline and three equally placed internal knots in the log scale; (b) an alternative four df was used in the cross-basis term for meteorological factors in the exposure-response function; (c) the maximum number of knots was reduced to 25 in the flexible natural cubic spline to control time trend in the tensor product smooths; (d) all demographic and socioeconomic variables were excluded from the model; (e) adjustment for the prevalence of smoking and obesity among adults was included in the model; (f) adjustment for climate zone was included in the model; (g) additional adjustment was made for the average PM_2.5_ concentration in each county during 2014–2018^45^; (h) additional adjustment was made for daily mean PM_2.5_, and daily maximum 8-h O_3_. For daily covariates with available data in only some of the counties or study period, the results of sensitivity analyses were compared to the main model re-run on the same partial dataset.

### Reporting summary

Further information on research design is available in the Nature Research Reporting Summary linked to this article.

## Supplementary information

Supplementary Information

Reporting Summary

## Data Availability

The data that support the findings of this study are available in GitHub (10.5281/zenodo.4766014)^72^.

## Code availability

R code for this analysis is available at https://github.com/CHENlab-Yale/COVID-Climate.
