# Supplementary material for: Role of meteorological factors in the transmission of SARS-CoV-2 in the United States
Source: Nat Commun. 2021 Jun 14;12:3602. doi: 10.1038/s41467-021-23866-7 (PMC8203661; doi:10.1038/s41467-021-23866-7)
Supplement: Supplementary file 1 — Supplementary Information [file 41467_2021_23866_MOESM1_ESM.pdf]

Supplementary Information

Role of meteorological factors in the transmission of SARS-CoV-2 in the United States

Yiqun Ma[1,2], Sen Pei[3], Jeffrey Shaman[3], Robert Dubrow[1,2], Kai Chen[1,2]

1 Department of Environmental Health Sciences, Yale School of Public Health, 60 College Street, New Haven, CT, 06520-8034, USA

2 Yale Center on Climate Change and Health, Yale School of Public Health, 60 College Street, New Haven, CT, 06520-8034, USA

3 Department of Environmental Health Sciences, Mailman School of Public Health, Columbia University, New York, NY 10032, USA.

Supplementary Notes. Equations used in the transmission model
The transmission dynamics are depicted by the following equations.

Daytime transmission:

$$S_{ij}(t+dt_1) = S_{ij}(t) - \frac{\beta_i S_{ij}(t)\sum_k I_{ki}^r(t)}{N_i^d(t)}dt_1 - \frac{\mu\beta_i S_{ij}(t)\sum_k I_{ik}^u(t)}{N_i^d(t)}dt_1 + \theta dt_1 \frac{N_{ij} - I_{ij}^r(t)}{N_i^d(t)} \sum_{k\neq i} \frac{\bar{N}_{ik}\sum_l S_{kl}(t)}{N_k^d(t) - \sum_l I_{lk}^r(t)} - \theta dt_1 \frac{S_{ij}(t)}{N_i^d(t) - \sum_l I_{li}^r(t)} \sum_{k\neq i} \bar{N}_{ki} \quad (1)$$

$$E_{ij}(t+dt_1) = E_{ij}(t) + \frac{\beta_i S_{ij}(t)\sum_k I_{ki}^r(t)}{N_i^d(t)}dt_1 + \frac{\mu\beta_i S_{ij}(t)\sum_k I_{ik}^u(t)}{N_i^d(t)}dt_1 - \frac{E_{ij}(t)}{Z}dt_1 + \theta dt_1 \frac{N_{ij} - I_{ij}^r(t)}{N_i^d(t)} \sum_{k\neq i} \frac{\bar{N}_{ik}\sum_l E_{kl}(t)}{N_k^d(t) - \sum_l I_{lk}^r(t)} - \theta dt_1 \frac{E_{ij}(t)}{N_i^d(t) - \sum_l I_{li}^r(t)} \sum_{k\neq i} \bar{N}_{ki} \quad (2)$$

$$I_{ij}^r(t+dt_1) = I_{ij}^r(t) + \alpha_i \frac{E_{ij}(t)}{Z}dt_1 - \frac{I_{ij}^r(t)}{D}dt_1 \quad (3)$$

$$I_{ij}^u(t+dt_1) = I_{ij}^u(t) + (1-\alpha_i)\frac{E_{ij}(t)}{Z}dt_1 - \frac{I_{ij}^u(t)}{D}dt_1 + \theta dt_1 \frac{N_{ij} - I_{ij}^r(t)}{N_i^d(t)} \sum_{k\neq i} \frac{\bar{N}_{ik}\sum_l I_{kl}^u(t)}{N_k^d(t) - \sum_l I_{lk}^r(t)} - \theta dt_1 \frac{I_{ij}^u(t)}{N_i^d(t) - \sum_l I_{li}^r(t)} \sum_{k\neq i} \bar{N}_{ki} \quad (4)$$

$$N_i^d(t) = N_{ii} + \sum_{k\neq i} I_{ki}^r(t) + \sum_{k\neq i}(N_{ik} - I_{ik}^r(t)) \quad (5)$$

Nighttime transmission:

$$S_{ij}(t+1) = S_{ij}(t+dt_1) - \frac{\beta_j S_{ij}(t+dt_1)\sum_k I_{kj}^r(t+dt_1)}{N_j^n}dt_2 - \frac{\mu\beta_j S_{ij}(t+dt_1)\sum_k I_{kj}^u(t+dt_1)}{N_j^n}dt_2 + \theta dt_2 \frac{N_{ij}}{N_j^n} \sum_{k\neq j} \frac{\bar{N}_{jk}\sum_l S_{lk}(t+dt_1)}{N_k^n - \sum_l I_{lk}^r(t+dt_1)} - \theta dt_2 \frac{S_{ij}(t+dt_1)}{N_j^n - \sum_k I_{kj}^r(t+dt_1)} \sum_{k\neq j} \bar{N}_{kj} \quad (6)$$

$$E_{ij}(t+1) = E_{ij}(t+dt_1) + \frac{\beta_j S_{ij}(t+dt_1)\sum_k I_{kj}^r(t+dt_1)}{N_j^n}dt_2 + \frac{\mu\beta_j S_{ij}(t+dt_1)\sum_k I_{kj}^u(t+dt_1)}{N_j^n}dt_2 - \frac{E_{ij}(t+dt_1)}{Z}dt_2 + \theta dt_2 \frac{N_{ij}}{N_j^n} \sum_{k\neq j} \frac{\bar{N}_{jk}\sum_l E_{lk}(t+dt_1)}{N_k^n - \sum_l I_{lk}^r(t+dt_1)} - \theta dt_2 \frac{E_{ij}(t+dt_1)}{N_j^n - \sum_k I_{kj}^r(t+dt_1)} \sum_{k\neq j} \bar{N}_{kj} \quad (7)$$

$$I_{ij}^r(t+1) = I_{ij}^r(t+dt_1) + \alpha_j \frac{E_{ij}(t+dt_1)}{Z}dt_2 - \frac{I_{ij}^r(t+dt_1)}{D}dt_2 \quad (8)$$

$$I_{ij}^u(t+1) = I_{ij}^u(t+dt_1) + (1-\alpha_j)\frac{E_{ij}(t+dt_1)}{Z}dt_2 - \frac{I_{ij}^u(t+dt_1)}{D}dt_2 + \theta dt_2 \frac{N_{ij}}{N_j^n} \sum_{k\neq j} \frac{\bar{N}_{jk}\sum_l I_{lk}^u(t+dt_1)}{N_k^n - \sum_l I_{lk}^r(t+dt_1)} - \theta dt_2 \frac{I_{ij}^u(t+dt_1)}{N_j^n - \sum_k I_{kj}^r(t+dt_1)} \sum_{k\neq j} \bar{N}_{kj} \quad (9)$$

$$N_i^n = \sum_k N_{ki} \quad (10)$$

Here, S_{ij}, E_{ij}, I_{ij}^r, I_{ij}^u and N_{ij} are the susceptible, exposed, reported infected, unreported infected and total populations in the subpopulation commuting from county j to county i ($i \leftarrow j$); β_i is the transmission rate of reported infections in county i; μ is the relative transmissibility of unreported infections; Z is the average latency period (from infection to contagiousness); D is the average duration of contagiousness; α_i is the fraction of documented infections in county i; θ is a multiplicative factor adjusting random movement; $\overline{N}_{ij} = (N_{ij} + N_{ji})/2$ is the average number of commuters between counties i and j; dt_1 and dt_2 are the durations of daytime and nighttime transmission; and N_i^d and N_i^n are the daytime and nighttime populations of county i. We assume the I_{ij}^r population is immobile and does not participate in human movement. We integrate Eqs. S1-S10 using a Poisson process to represent the stochasticity of the transmission process.

Supplementary Table 1. Summary of R_t, daily cases, meteorological variables, and other county-level characteristics in 2,669 U.S. counties

	Mean (SD)	Min	P_{25}	Median	P_{75}	Max
**Daily records (March 15 to December 31, 2020)**						
R_t	1.49 (0.65)	0.45	1.06	1.43	1.81	6.62
Cases (count)	26.03 (139.84)	0	0	3	15	28,408
Temperature (°C)	16.52 (9.24)	-22.25	9.82	18.04	24.17	39.98
Specific humidity (g kg^{-1})	9.35 (4.96)	0.49	4.93	8.67	13.58	22.37
Ultraviolet radiation (kJ m^{-2})	81.97 (36.61)	1.68	50.60	86.29	113.36	155.61
**County characteristics**						
Population density (people per square mile)	310.27 (1947.37)	0.80	25.40	55.67	144.37	72041.02
Black residents (%)	10.01 (15.00)	0.00	0.87	2.94	12.14	87.41
Hispanic residents (%)	9.20 (13.58)	0.00	2.20	4.16	9.45	99.07
People aged 60+ (%)	26.03 (5.22)	7.09	22.73	25.88	28.82	66.63
Median household income ($)	51,743 (13,683)	20,188	42,689	50,037	57,899	136,268
Owner-occupied housing (%)	71.91 (7.62)	19.26	68.32	73.23	76.93	89.66
Residents over 25 years old without a high school diploma (%)	13.62 (6.19)	2.02	9.02	12.32	17.40	48.52
Healthcare workers (%)	2.63 (0.80)	0.12	2.10	2.63	3.14	9.48
ICU beds (per 10,000 people)	0.14 (0.20)	0.00	0.00	0.09	0.22	2.29

SD: standard deviation; Min: minimum; P25: 25th percentile; P75: 75th percentile; Max: maximum

R_t: reproduction number

Supplementary Table 2. Coefficient table of covariates in the main model

Covariates	Percent change in R_t[a]	95% CI	*P* value
Population density	-0.36	(-1.30, 0.59)	0.461
Black residents	-2.60	(-4.07, -1.10)	0.001
Hispanic residents	1.72	(-0.45, 3.94)	0.121
People aged 60+	-4.11	(-5.32, -2.88)	0.000
Household income	-2.11	(-3.72, -0.47)	0.012
House owner	-0.11	(-1.56, 1.36)	0.884
Education	1.71	(-0.06, 3.50)	0.058
ICU beds	1.22	(0.24, 2.21)	0.015
Healthcare workers	1.96	(0.89, 3.05)	0.000
Day when 100 cases/ 100,000 people was reached[b]	2.28	(1.14, 3.44)	0.000

This table displays the coefficients of covariates estimated by the generalized additive mixed model. The *P* values are two-sided.

R_t: reproduction number

ICU: intensive care unit

[a]percent change in R_t associated with 1 standard deviation increase of each covariate

[b]the date when cumulative confirmed COVID-19 cases reached 100 cases/100,000 people in each county; included as a number in the model

Supplementary Table 3. Performance of the main model

Model statistics	Value
R^2	0.51
Variance explained by the random effect of county	29.9%
Variance explained by the random effect of state	1.0%
Spatial autocorrelation (*P* value)[a]	0.16
Temporal autocorrelation (*P* value)[b]	0.80
REML	204030

This table displays the diagnostic statistics of the generalized additive mixed model.
REML: restricted maximum likelihood
[a]measured by Moran's I test, two-sided
[b]measured by Durbin-Watson test, two-sided

Supplementary Table 4. Sensitivity analyses for attributable fraction of R_t (%, eCI) across all 2,669 counties over the entire study period

	Air temperature	Specific humidity	Ultraviolet radiation
**Main model**	**3.73 (3.66, 3.76)**	**9.35 (9.27, 9.39)**	**4.44 (4.38, 4.47)**
Adjust for or exclude county-level factors [a]			
Exclude socioeconomic factors	3.05 (2.93, 3.11)	9.62 (9.55, 9.67)	3.71 (3.65, 3.75)
Adjust for smoking and obesity prevalence	3.78 (3.71, 3.82)	9.32 (9.25, 9.37)	4.43 (4.37, 4.46)
Adjust for long-term $PM_{2.5}$	5.15 (5.09, 5.19)	9.71 (9.64, 9.75)	3.36 (3.31, 3.40)
Adjust for climate zone	3.74 (3.68, 3.79)	9.32 (9.25, 9.36)	4.43 (4.38, 4.47)
Adjust for daily $PM_{2.5}$ ***in counties with available data*** [b]			
Main model (without $PM_{2.5}$)	15.63 (15.42, 15.76)	4.94 (4.81, 5.03)	3.30 (3.23, 3.35)
Adjust for daily $PM_{2.5}$	16.51 (16.27, 16.64)	4.20 (4.05, 4.28)	3.74 (3.65, 3.80)
Adjust for daily O_3 ***in counties with available data*** [c]			
Main model (without O_3)	6.29 (6.04, 6.43)	7.78 (7.47, 7.94)	5.17 (4.92, 5.35)
Adjust for daily O_3	6.77 (6.51, 6.92)	6.07 (5.74, 6.24)	6.28 (6.01, 6.44)

R_t: reproduction number

eCI: empirical confidence interval

[a] using full data with the same counties and period as in the main analysis.

[b] using data in counties with available daily $PM_{2.5}$ data (773 counties).

[c] using data in counties with available daily O_3 data (771 counties).

Supplementary Table 5. Spearman correlation coefficients among main daily variables

	R_t	Daily cases	Temperature	SH	UV radiation
R_t	1.00				
Daily cases	0.12	1.00			
Temperature	-0.18	-0.10	1.00		
SH	-0.09	-0.09	0.89	1.00	
UV radiation	-0.27	-0.29	0.57	0.37	1.00

R_t: reproduction number
SH: specific humidity
UV: ultraviolet

Supplementary Table 6. Spearman correlation coefficients among county-level mean R_t and other characteristics

	R_t	Population density	Black residents	Hispanic residents	People aged 60+	Household income	House owner	Education	Healthcare workers	ICU beds
R_t	1.00									
Population density	0.36	1.00								
Black residents	0.23	0.41	1.00							
Hispanic residents	-0.11	0.15	0.13	1.00						
People aged 60+	-0.14	-0.38	-0.32	-0.39	1.00					
Household income	-0.13	0.34	-0.18	0.27	-0.22	1.00				
House owner	-0.03	-0.28	-0.32	-0.36	0.45	0.10	1.00			
Education	0.17	-0.17	0.32	0.08	-0.11	-0.66	-0.09	1.00		
Healthcare workers	0.05	0.34	-0.03	-0.23	0.06	0.35	0.03	-0.49	1.00	
ICU beds	0.13	0.52	0.24	0.15	-0.21	0.07	-0.45	-0.12	0.30	1.00

R_t: reproduction number
ICU: intensive care unit

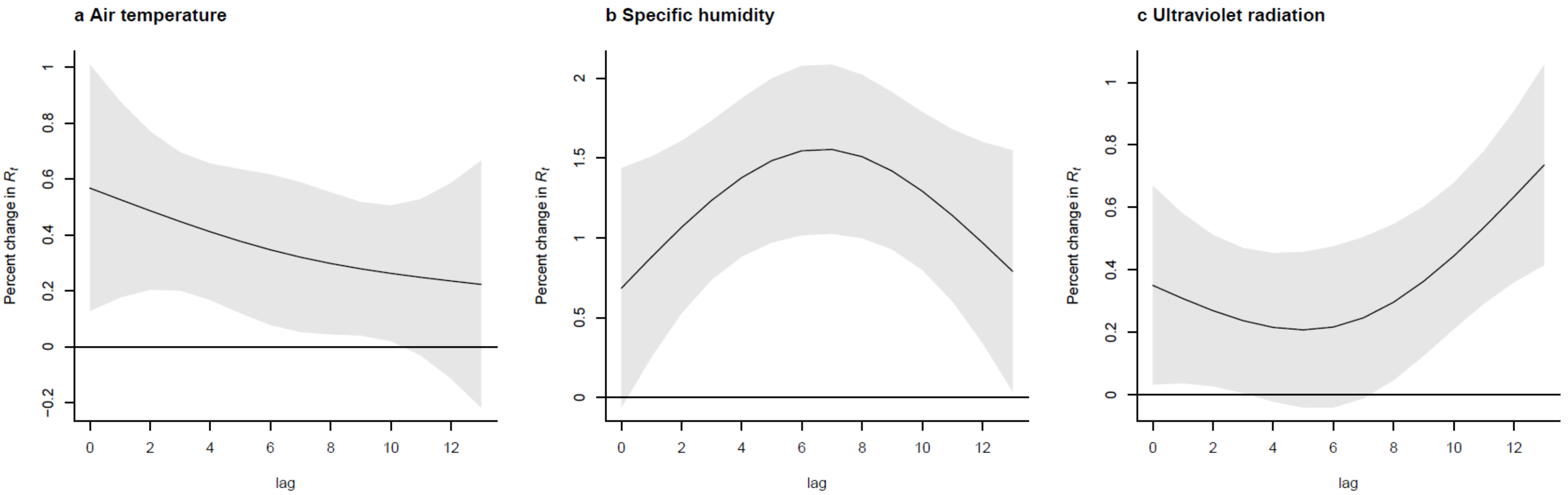

Supplementary Fig. 1. Lag-response relationships of air temperature, specific humidity, and ultraviolet radiation with reproduction number (*R_t*)

These curves are computed for the air temperature of 20 °C (**a**), the 1st percentile of specific humidity (**b**), and the ultraviolet (UV) radiation of 100 kJ m^{-2} (**c**) vs. the optimum values on different lag days; the grey areas display the 95% confidence interval. The effect estimates of temperature show a decreasing trend in the lag dimension, diminishing to 0.2% around lag day 13; the effect estimates of specific humidity show an inverted U-shaped trend in the lag dimension, with the smallest effects (0.7-0.8%) on lag days 0 and 13; the effect estimates of UV radiation decreases from the current day to lag day 6, and increases from lag day 6 to 13.

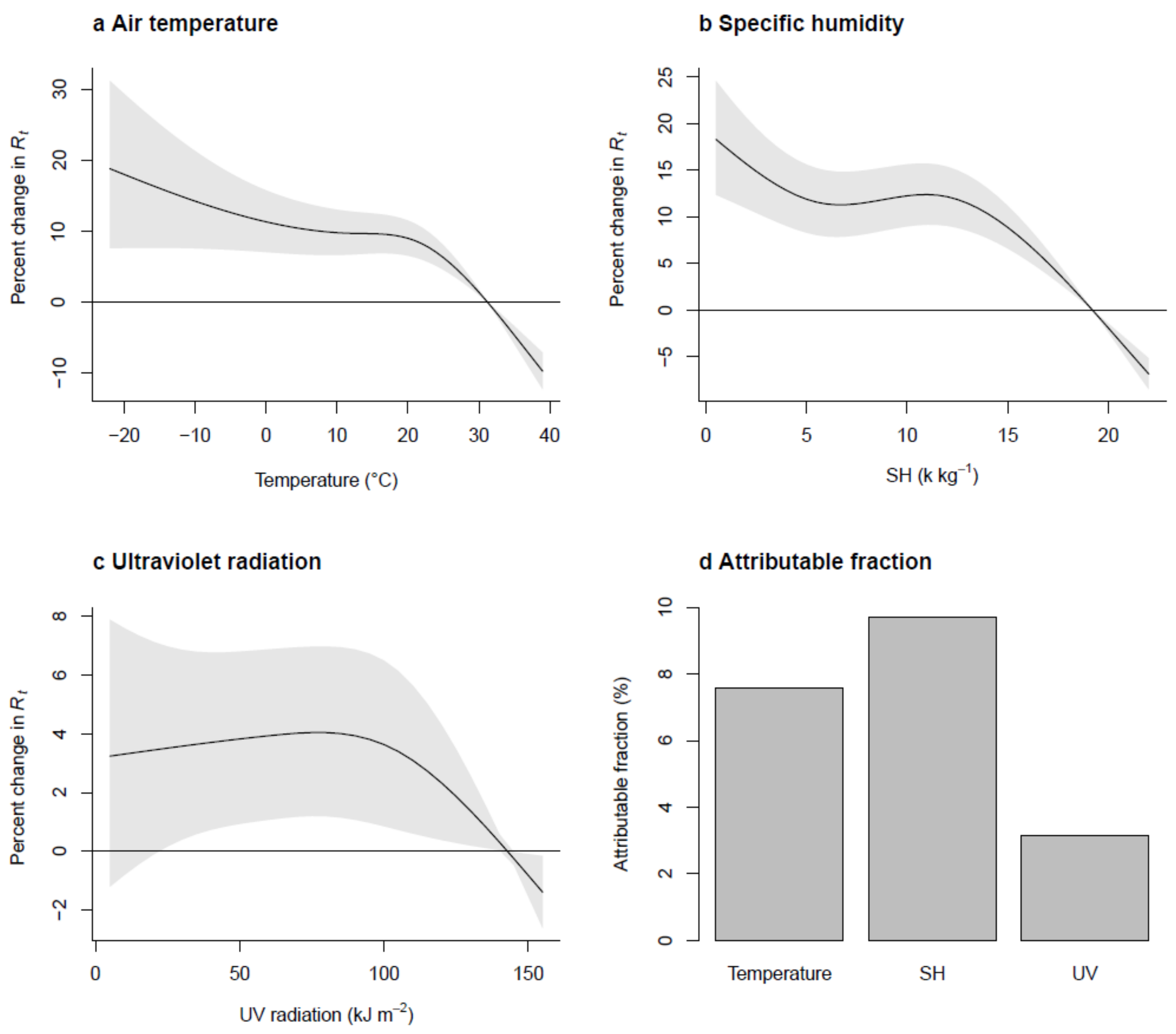

Supplementary Fig. 2. Results from separate models for each meteorological factor

This figure shows the results from separate models for air temperature, specific humidity, and ultraviolet radiation, respectively. **a, b, c** The estimated exposure-response curves for the associations of air temperature, specific humidity (SH), and ultraviolet (UV) radiation with reproduction number (R_t) for SARS-Cov-2. Grey area: 95% confidence interval. **d** The fraction of reproduction number (R_t) attributable to temperature, SH, and UV radiation. Note that different from the results from main model, these attributable fractions cannot be summed up.

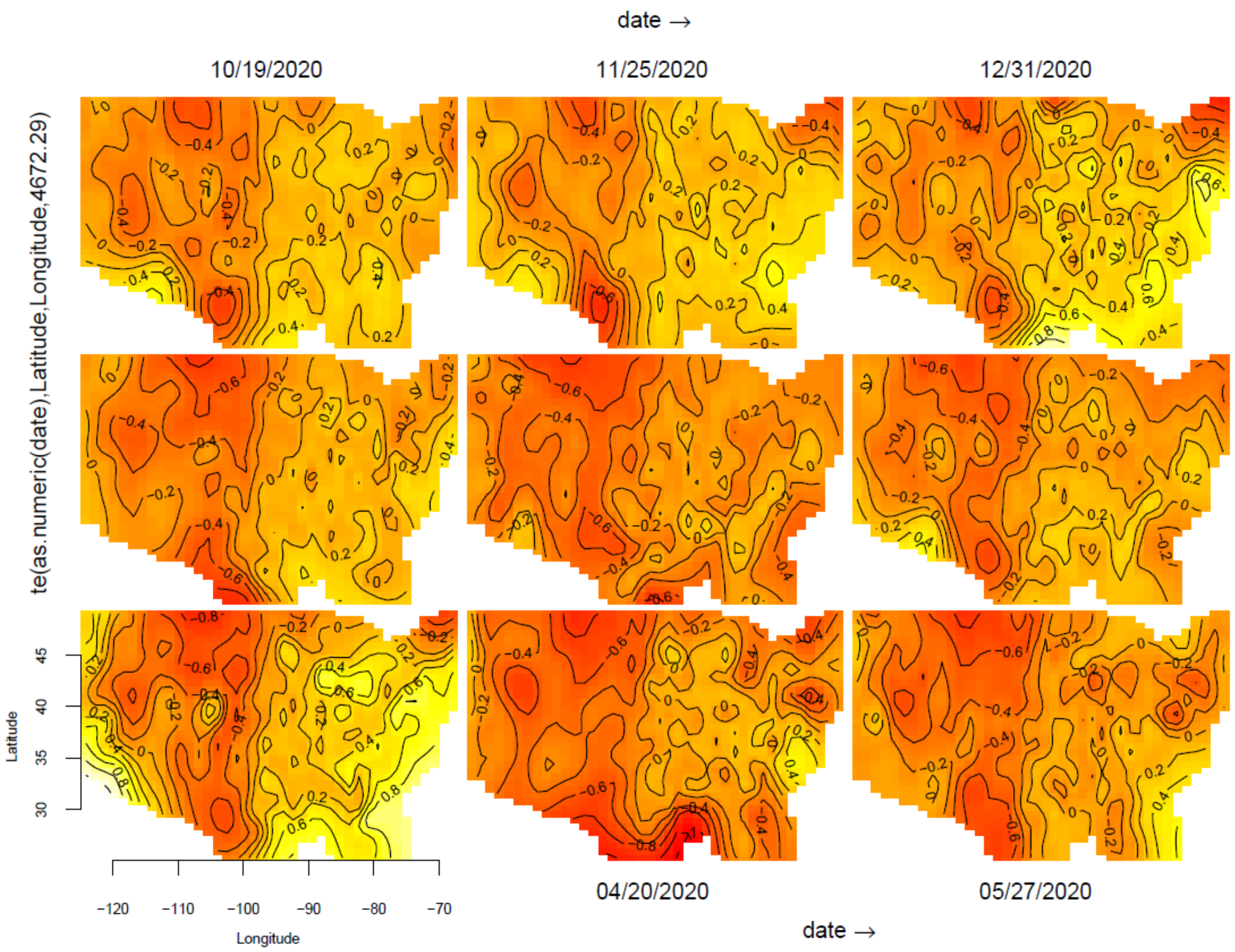

Supplementary Fig. 3. Plot of the tensor product smooth in the main model

In the main model, the time trend was controlled by a flexible natural cubic spline over the range of study dates with a maximum of 30 knots; a thin plate spline with a maximum of 200 knots was used to control the coordinates of the centroid of each county. We constructed tensor product smooths of the splines of geographical coordinates and time, to better control for the temporal and spatial variations. Darker orange represents higher effect estimates of the smooths, and lighter orange represents lower effect estimates.

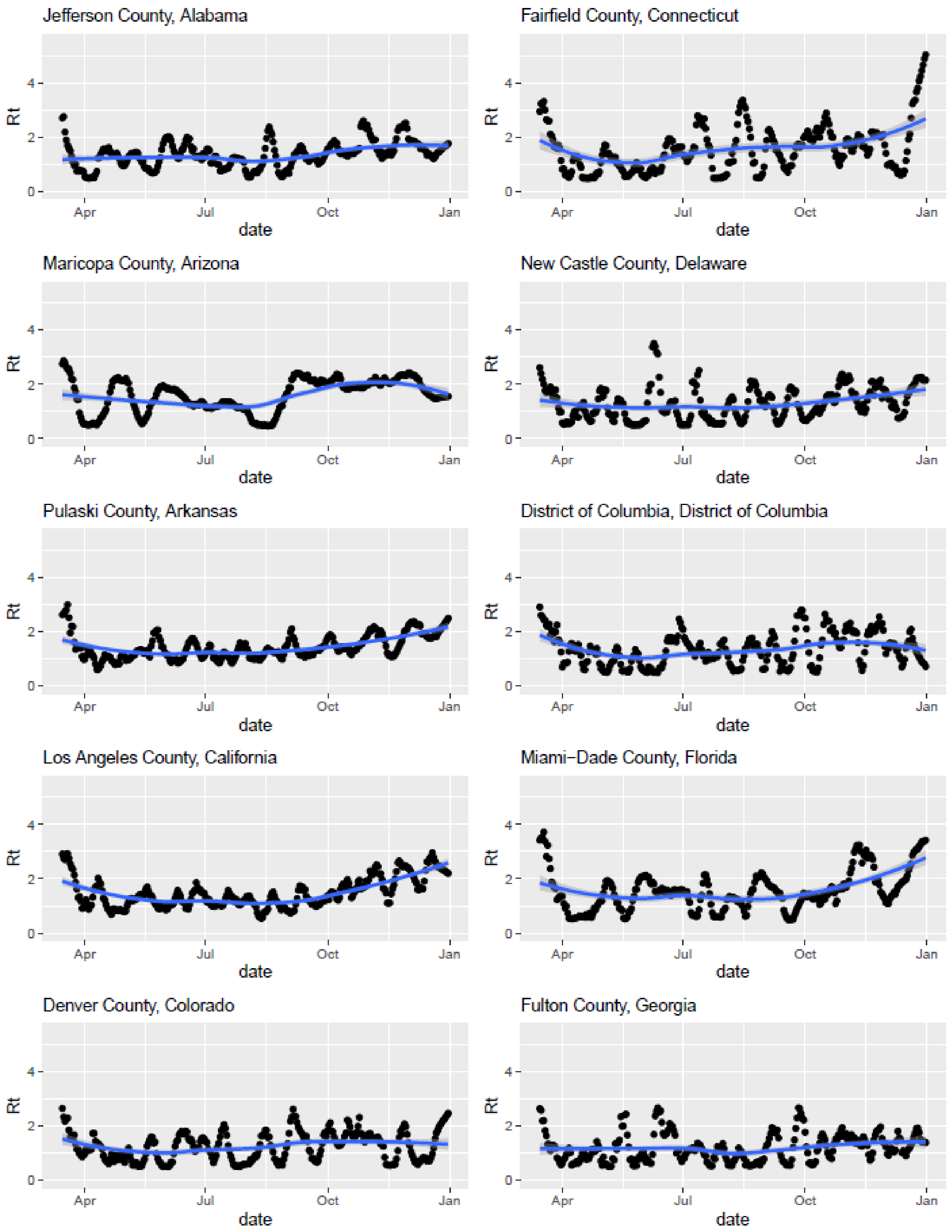
Jefferson County, Alabama
Fairfield County, Connecticut
Maricopa County, Arizona
New Castle County, Delaware
Pulaski County, Arkansas
District of Columbia, District of Columbia
Los Angeles County, California
Miami−Dade County, Florida
Denver County, Colorado
Fulton County, Georgia
Rt
0
2
4
Apr
Jul
Oct
Jan
date

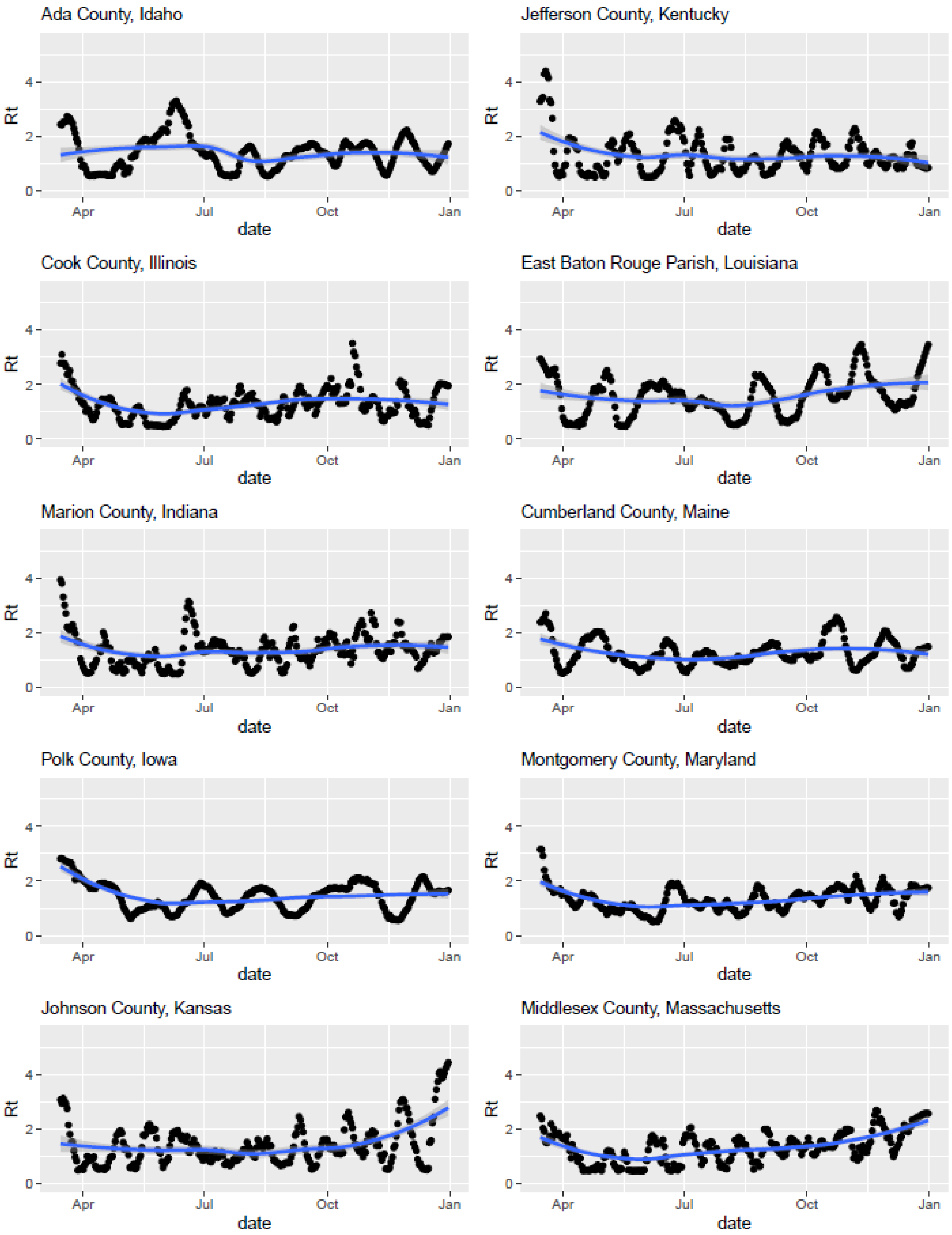
Ada County, Idaho
Jefferson County, Kentucky
Cook County, Illinois
East Baton Rouge Parish, Louisiana
Marion County, Indiana
Cumberland County, Maine
Polk County, Iowa
Montgomery County, Maryland
Johnson County, Kansas
Middlesex County, Massachusetts
Rt
0
2
4
Apr
Jul
Oct
Jan
date

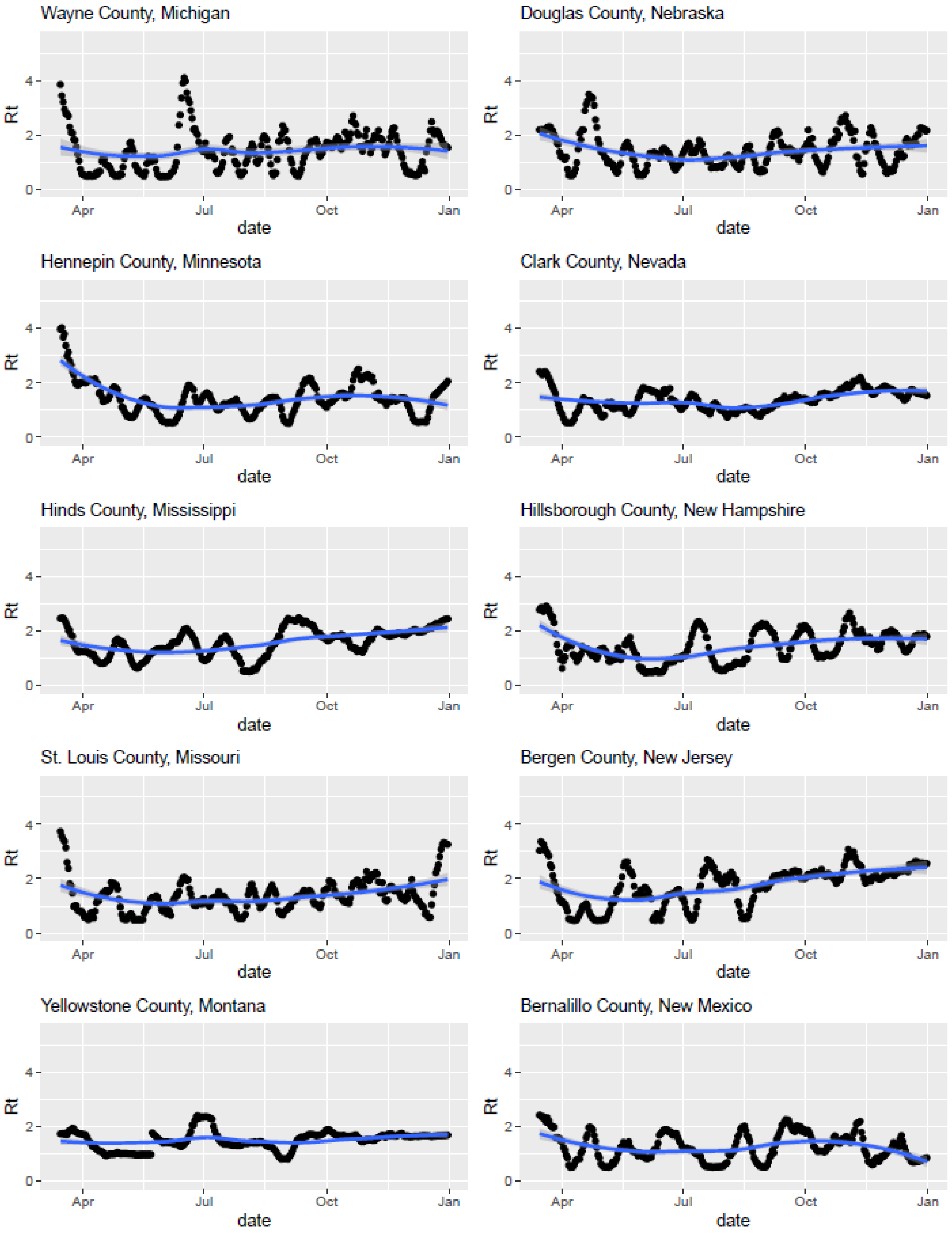
Wayne County, Michigan
Douglas County, Nebraska
Hennepin County, Minnesota
Clark County, Nevada
Hinds County, Mississippi
Hillsborough County, New Hampshire
St. Louis County, Missouri
Bergen County, New Jersey
Yellowstone County, Montana
Bernalillo County, New Mexico
Rt
0
2
4
Apr
Jul
Oct
Jan
date

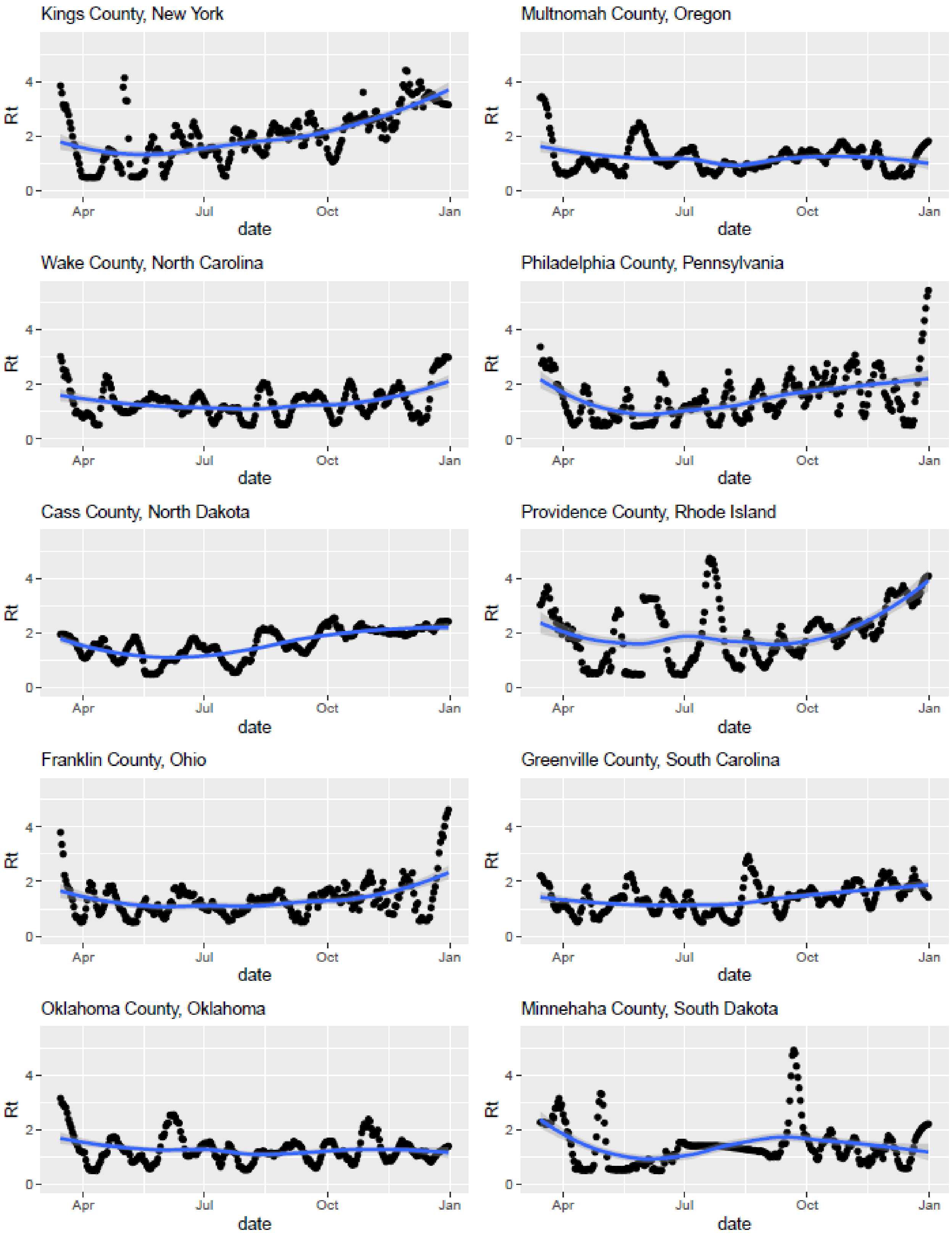
Kings County, New York
Multnomah County, Oregon
Wake County, North Carolina
Philadelphia County, Pennsylvania
Cass County, North Dakota
Providence County, Rhode Island
Franklin County, Ohio
Greenville County, South Carolina
Oklahoma County, Oklahoma
Minnehaha County, South Dakota
Rt
0
2
4
Apr
Jul
Oct
Jan
date

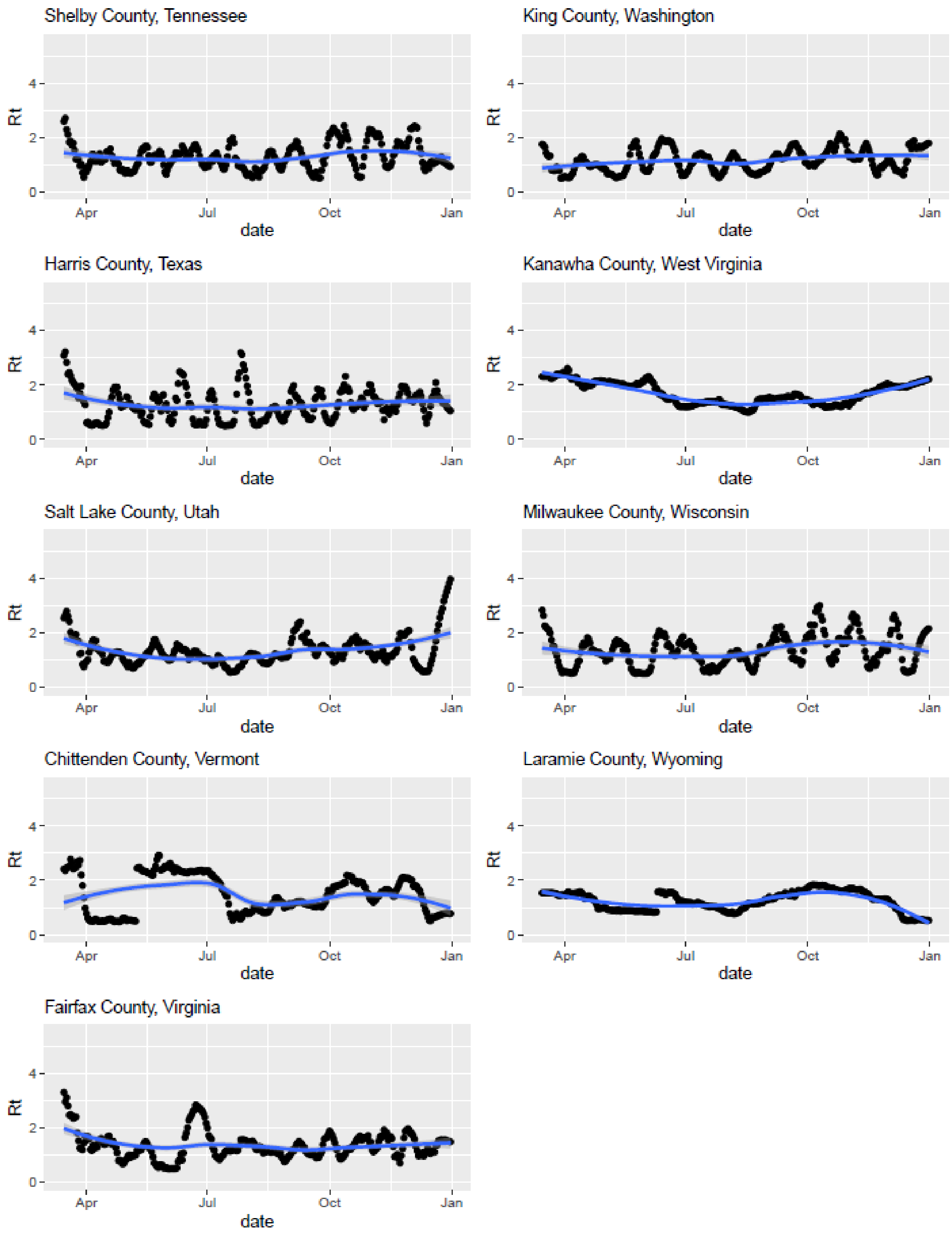

Supplementary Fig. 4. Daily R_t from March 15 to December 31, 2020 in the largest county in each state

Black dots represent the daily value of reproduction number (R_t) in the largest county in each U.S. state. Blue lines show the trend of R_t through time, fitted by local polynomial regression; the light blue areas display the 95% confidence interval.

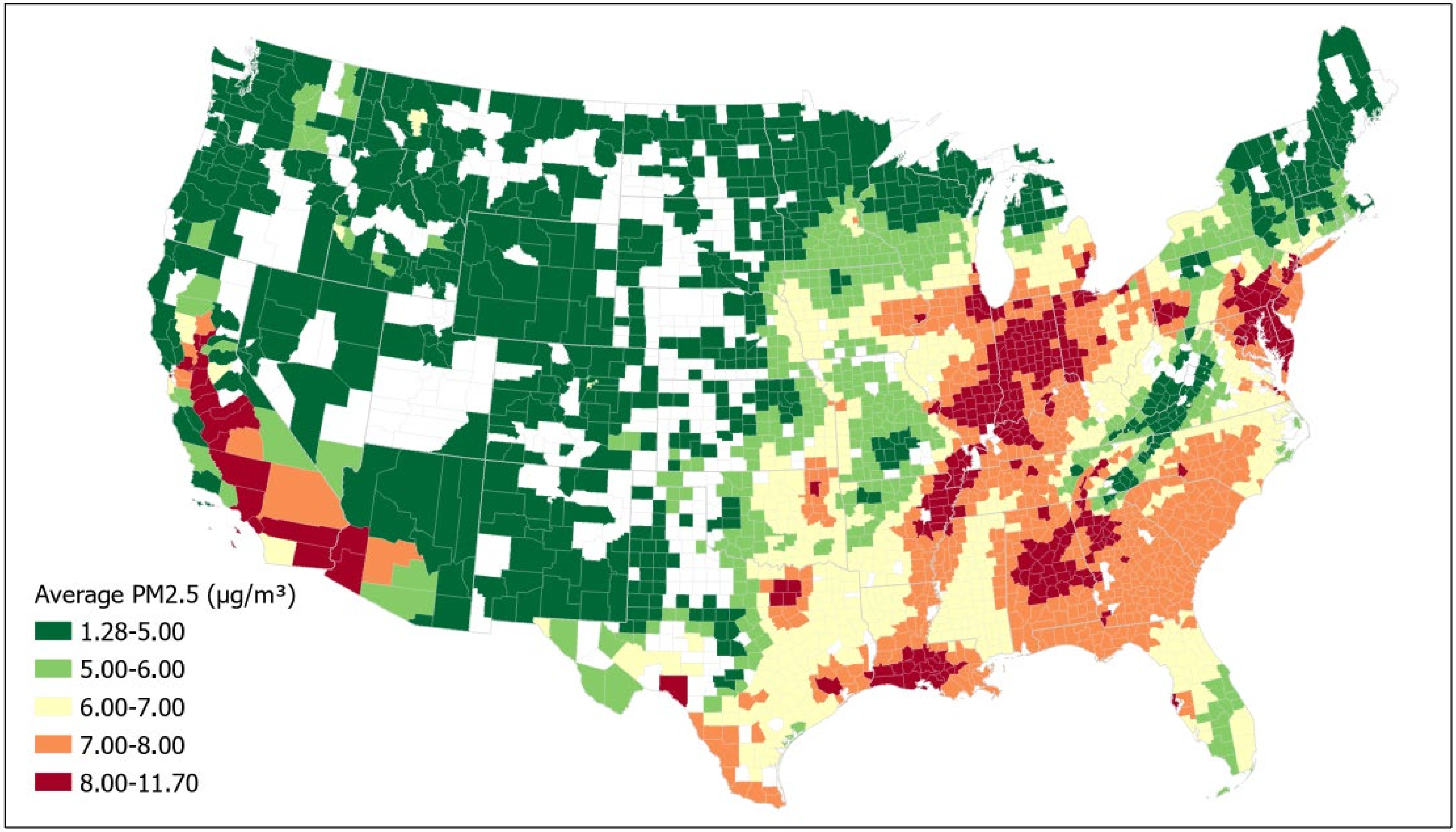

Supplementary Fig. 5. Distribution of average $PM_{2.5}$ concentration during 2014-2018

This map displays the county-level average $PM_{2.5}$ concentration during 2014-2018, extracted from the $PM_{2.5}$ estimation provided by Atmospheric Composition Analysis Group[1]. The shapefile in the map was obtained from the U.S. Census Bureau.

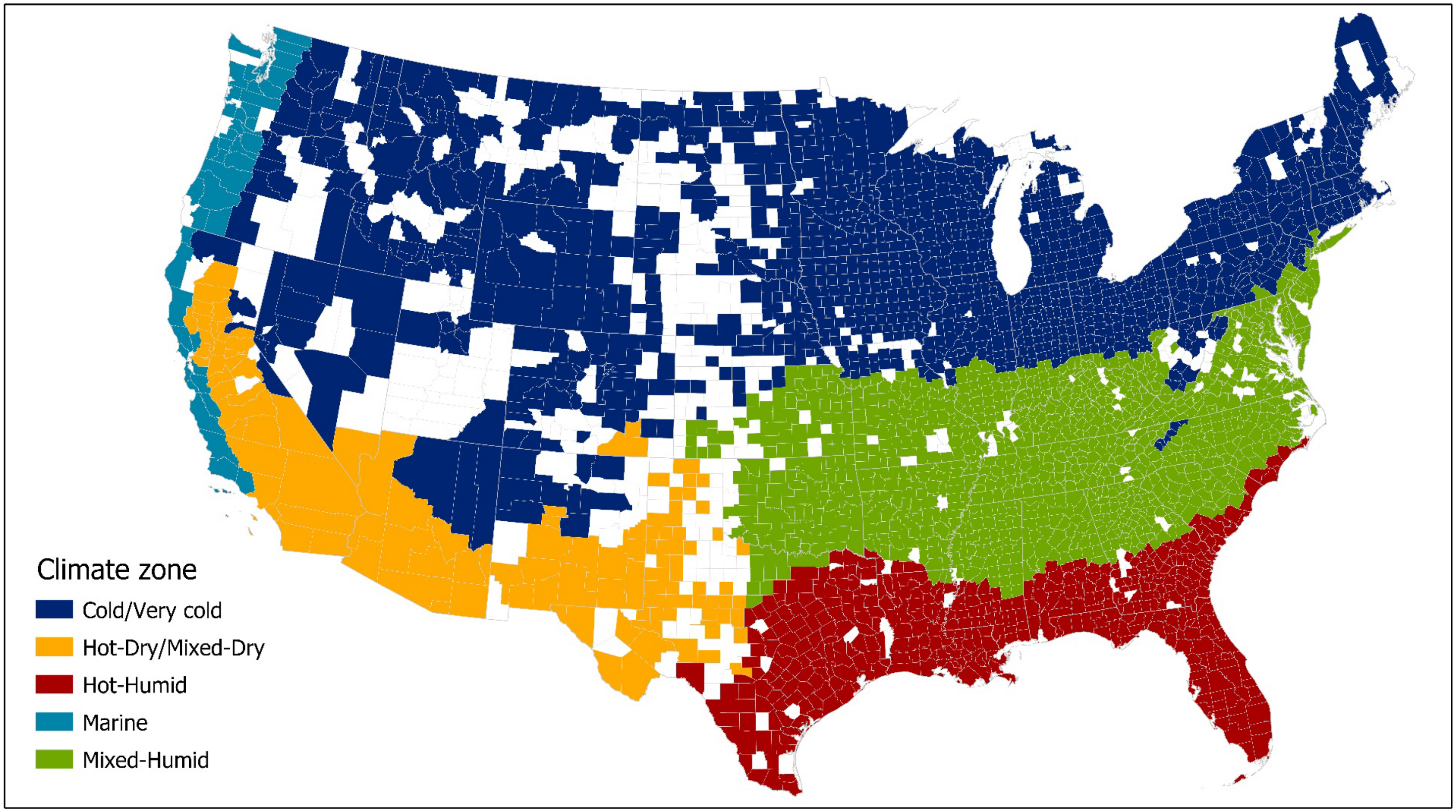

Supplementary Fig. 6. Distribution of climate zones of the 2,669 U.S. counties

This map displays the distribution of the climate zones of the 2,669 U.S. counties. We categorized study counties into one of five climate zones based on the guide released by U.S. Department of Energy[2]. The shapefile in the map was obtained from the U.S. Census Bureau.

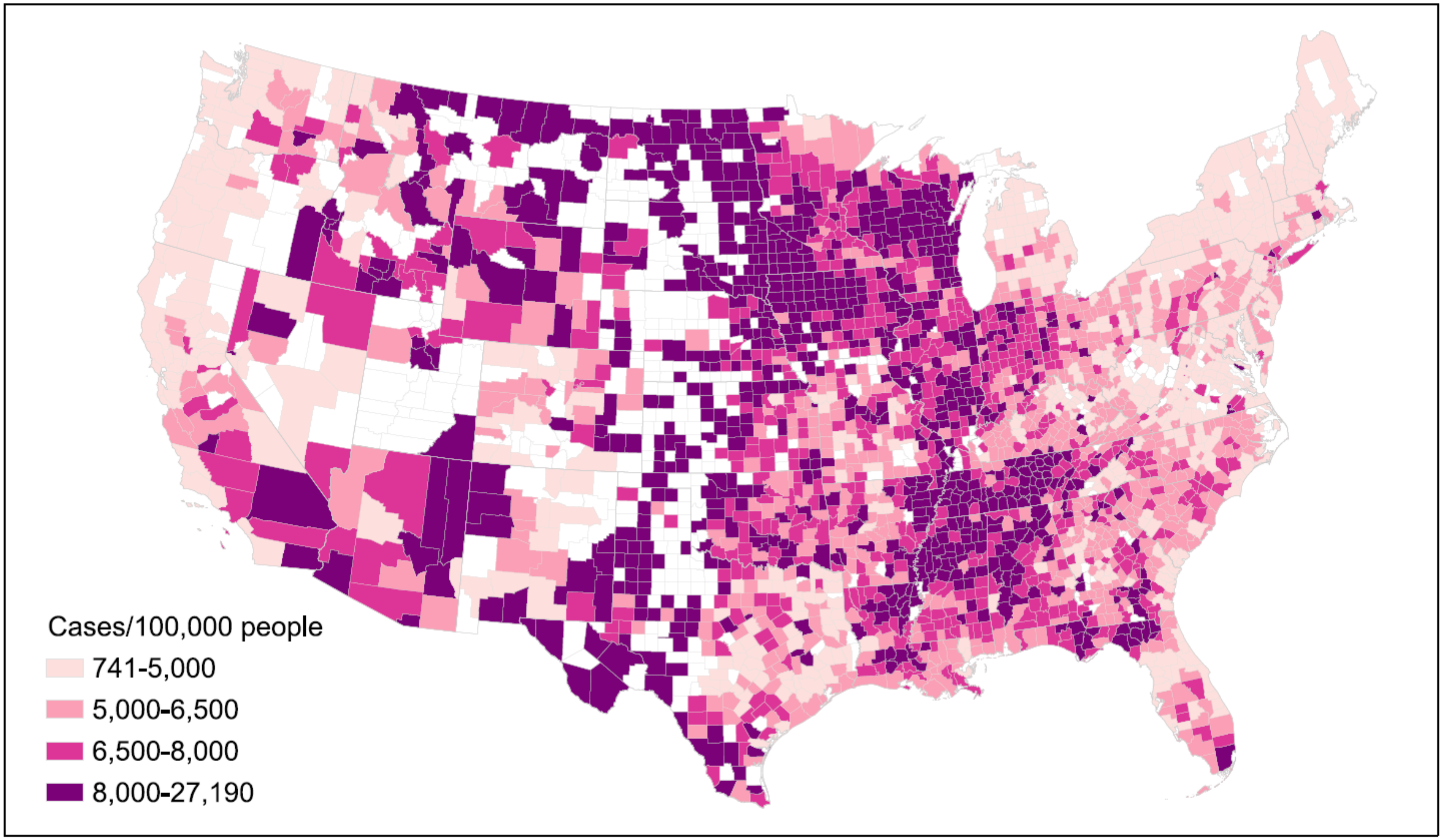

Supplementary Fig. 7. Map of cumulative cases in the 2,669 U.S. counties up to December 31, 2020

This map displays the distribution of the cumulative cases per 100,000 population in the 2,669 U.S. counties, up to December 31, 2020, based on the public datasets provided by John Hopkins University[3]. The shapefile in the map was obtained from the U.S. Census Bureau.

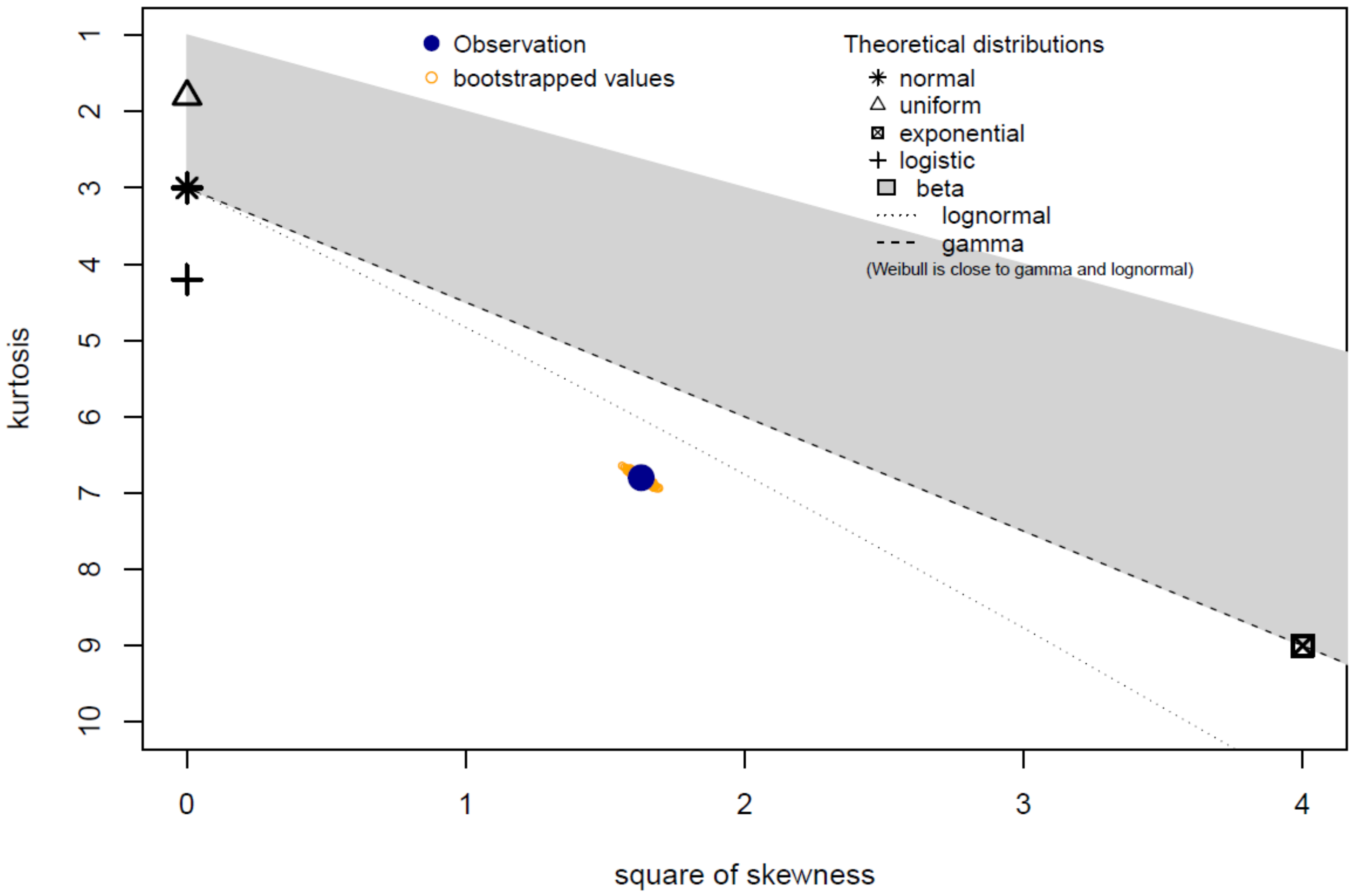

Supplementary Fig. 8. Skewness-kurtosis plot for distribution of daily reproduction number

A skewness-kurtosis plot such as the one proposed by Cullen and Frey (1999)[4] was generated for the empirical distribution of daily reproduction number in our data. To account for the uncertainty of the estimated values of kurtosis and skewness from data, 1000 bootstrap samples were generated, and the computed values were reported in orange dots on the plot. Skewness and Pearsons's kurtosis values for common distributions were displayed as a tool to help choose the distribution that the data best fit. The distribution of daily reproduction number was closest to the lognormal distribution.

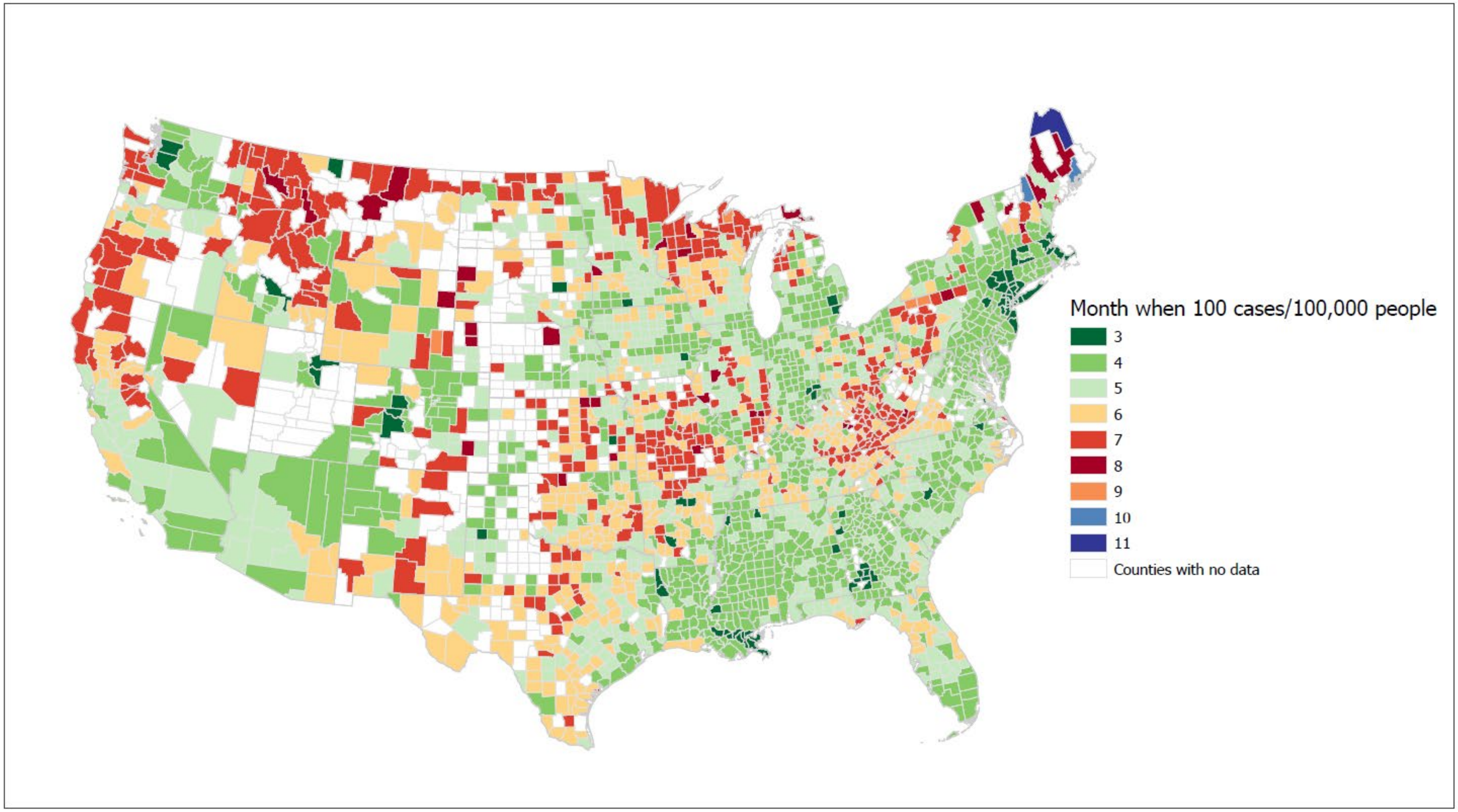

Supplementary Fig. 9. Month when cumulative confirmed COVID-19 cases reached 100 cases per 100,000 people in each county
This map displays the month in 2020 in which the cumulative confirmed COVID-19 cases reached 100 cases per 100,000 people in each study county. The shapefile in the map was obtained from the U.S. Census Bureau.

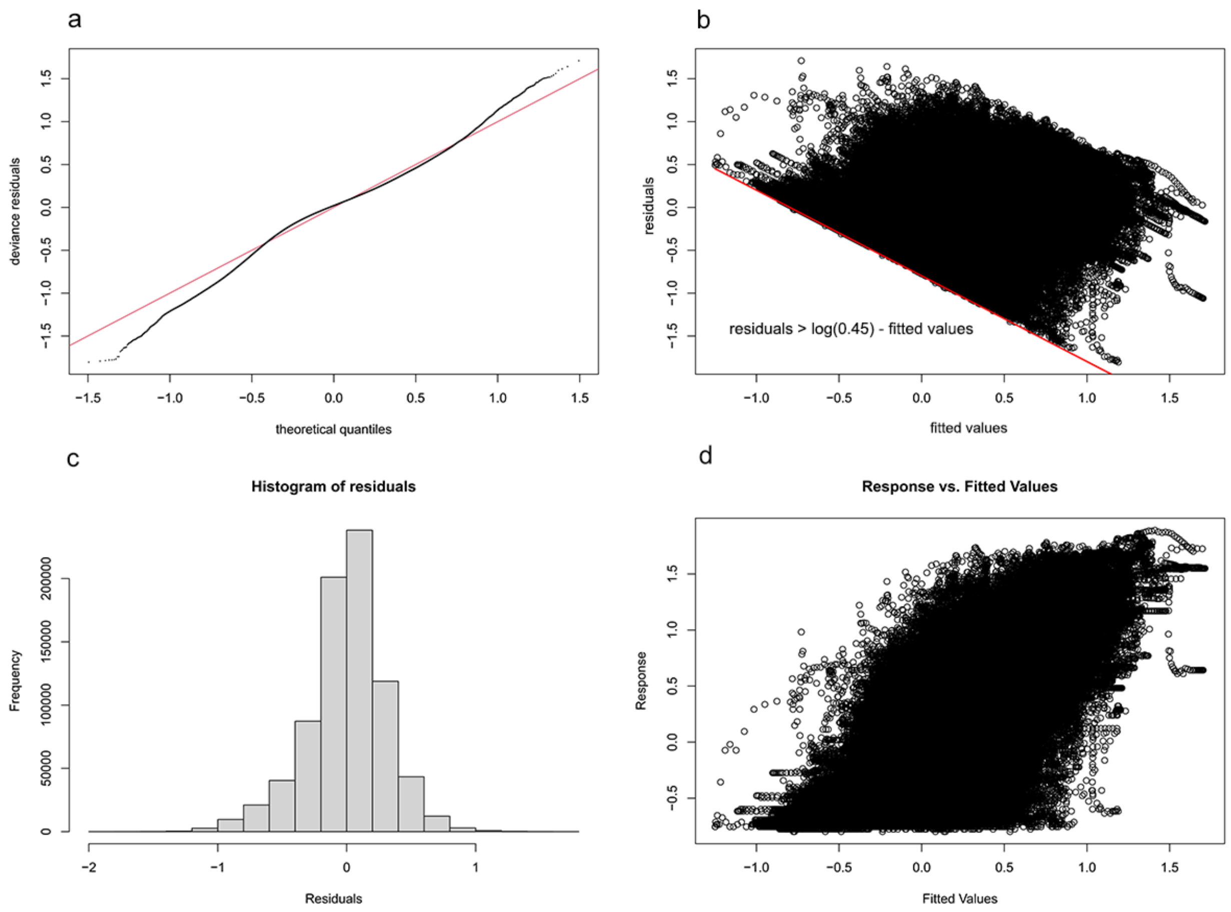

Supplementary Fig. 10. Diagnostic plots for the main model

This figure displays the four diagnostic plots for the main model. **a** The Q-Q plot shows deviance residuals against approximate theoretical quantiles of the deviance residual distribution. The residuals are generally normally distributed, although slightly heavy tailed. **b** This plot indicates no clear pattern in the residual vs. fitted values, except for a line of residuals at the lowest values (the red line), which corresponds to the minimum R_t in our dataset (log(0.45) in the y-axis). **c** The histogram of residuals indicates that the residuals are generally normally distributed. **d** This plot shows response variable (log(R_t)) in the model against fitted values (log(R_t) fitted by the model). This plot indicates that the actual R_t and the fitted R_t generally match up, except for the response values with a minimum value of natural log of 0.45 (i.e., -0.8).

Supplementary References

1. van Donkelaar, A., Martin, R.V., Li, C. & Burnett, R.T. Regional estimates of chemical composition of fine particulate matter using a combined geoscience-statistical method with information from satellites, models, and monitors. *Environ Sci Technol* **53**, 2595-2611 (2019).
2. U.S. Department of Energy. Guide to Determining Climate Regions by County. vol. 7.3. (2015).
3. Dong, E., Du, H. & Gardner, L. An interactive web-based dashboard to track COVID-19 in real time. *Lancet Infect Dis* **20**, 533-534 (2020).
4. Cullen, A.C., Frey, H.C. & Frey, C.H. *Probabilistic Techniques in Exposure Assessment: A Handbook for Dealing with Variability and Uncertainty in Models and Inputs*, (Springer Science & Business Media, 1999).
