# Supplementary material for: Role of meteorological factors in the transmission of SARS-CoV-2 in the United States
Source: Nat Commun. 2021 Jun 14;12:3602. doi: 10.1038/s41467-021-23866-7 (PMC8203661; doi:10.1038/s41467-021-23866-7)
Supplement: Supplementary file 2 — Reporting Summary [file 41467_2021_23866_MOESM2_ESM.pdf]

nature research

Corresponding author(s): Kai Chen

Last updated by author(s): May 16, 2021

Reporting Summary

Nature Research wishes to improve the reproducibility of the work that we publish. This form provides structure for consistency and transparency in reporting. For further information on Nature Research policies, see our Editorial Policies and the Editorial Policy Checklist.

Statistics

For all statistical analyses, confirm that the following items are present in the figure legend, table legend, main text, or Methods section.

n/a	Confirmed	
☐	☒	The exact sample size (n) for each experimental group/condition, given as a discrete number and unit of measurement
☒	☐	A statement on whether measurements were taken from distinct samples or whether the same sample was measured repeatedly
☐	☒	The statistical test(s) used AND whether they are one- or two-sided<br>*Only common tests should be described solely by name; describe more complex techniques in the Methods section.*
☐	☒	A description of all covariates tested
☐	☒	A description of any assumptions or corrections, such as tests of normality and adjustment for multiple comparisons
☐	☒	A full description of the statistical parameters including central tendency (e.g. means) or other basic estimates (e.g. regression coefficient) AND variation (e.g. standard deviation) or associated estimates of uncertainty (e.g. confidence intervals)
☐	☒	For null hypothesis testing, the test statistic (e.g. F, t, r) with confidence intervals, effect sizes, degrees of freedom and P value noted<br>*Give P values as exact values whenever suitable.*
☒	☐	For Bayesian analysis, information on the choice of priors and Markov chain Monte Carlo settings
☐	☒	For hierarchical and complex designs, identification of the appropriate level for tests and full reporting of outcomes
☐	☒	Estimates of effect sizes (e.g. Cohen's d, Pearson's r), indicating how they were calculated

Our web collection on statistics for biologists contains articles on many of the points above.

Software and code

Policy information about availability of computer code

Data collection	R code for this analysis is available at https://github.com/CHENlab-Yale/COVID-Climate
Data analysis	All statistical analyses were conducted with R software (version 3.6.1) using the mgcv (version 1.8-33) and dlnm (version 2.4.2) package.

For manuscripts utilizing custom algorithms or software that are central to the research but not yet described in published literature, software must be made available to editors and reviewers. We strongly encourage code deposition in a community repository (e.g. GitHub). See the Nature Research guidelines for submitting code & software for further information.

Data

Policy information about availability of data

All manuscripts must include a data availability statement. This statement should provide the following information, where applicable:

- Accession codes, unique identifiers, or web links for publicly available datasets
- A list of figures that have associated raw data
- A description of any restrictions on data availability

The data that support the findings of this study are available in GitHub (DOI: 10.5281/zenodo.4766014)

Field-specific reporting

Life sciences study design

All studies must disclose on these points even when the disclosure is negative.

Sample size	This study intended to cover all counties in the U.S., but we excluded counties with less than 400 cumulative confirmed cases as of December 31, 2020 to avoid possible inaccurate estimation for counties with few cases. Finally, this study included daily records of meteorological variables and reproduction number for a total of 2669 U.S. counties, from March 15 to December 31, 2020.<br>The final sample size has 779348 records, covering 88.8% of the U.S. counties and 292 days in 2020. Therefore, the sample size is sufficient.
Data exclusions	To avoid possible inaccurate estimation for counties with few cases, we inferred Rt in the 2,669 U.S. counties with at least 400 cumulative confirmed cases as of December 31, 2020.
Replication	This is an observational study using reported COVID-19 epidemiological data and meteorological data, combined with other covariates. The results are solely produced by statistical analyses. All data and code are provided in GitHub, and the results can be replicated. In addition, when analyzing the data, the authors have run the model for multiple times and got the same results. Sensitivity analyses have also been conducted and the results were similar to those from the main model.
Randomization	There was no experimental design or group allocation involved in this study
Blinding	There was no experimental design or group allocation involved in this study

Reporting for specific materials, systems and methods

We require information from authors about some types of materials, experimental systems and methods used in many studies. Here, indicate whether each material, system or method listed is relevant to your study. If you are not sure if a list item applies to your research, read the appropriate section before selecting a response.

Materials & experimental systems

n/a	Involved in the study	
☒	☐	Antibodies
☒	☐	Eukaryotic cell lines
☒	☐	Palaeontology and archaeology
☒	☐	Animals and other organisms
☒	☐	Human research participants
☒	☐	Clinical data
☒	☐	Dual use research of concern

Methods

n/a	Involved in the study	
☒	☐	ChIP-seq
☒	☐	Flow cytometry
☒	☐	MRI-based neuroimaging
